# In situ adsorption of itaconic acid from fermentations of *Ustilago cynodontis* improves bioprocess efficiency

**DOI:** 10.1186/s13068-023-02433-w

**Published:** 2023-11-26

**Authors:** Johannes Pastoors, Alexander Deitert, Carina Michel, Karsten Günster, Maurice Finger, Jordy Hofstede, Jeff Deischter, Andreas Biselli, Jörn Viell, Regina Palkovits, Andreas Jupke, Jochen Büchs

**Affiliations:** 1 AVT—Biochemical Engineering, RWTH Aachen University, Forckenbeckstraße 51, 52074 Aachen, Germany; 2 AVT—Process Systems Engineering, RWTH Aachen University, Forckenbeckstraße 51, 52074 Aachen, Germany; 3 ITMC—Institute of Technical and Macromolecular Chemistry, RWTH Aachen University, Worringerweg 2, 52074 Aachen, Germany; 4 AVT—Fluid Process Engineering, RWTH Aachen University, Forckenbeckstraße 51, 52074 Aachen, Germany

**Keywords:** Adsorption, Raman spectroscopy, Downstream processing, Integrated bioprocesses, Itaconic acid, Biorefinery processes

## Abstract

**Background:**

Reducing the costs of biorefinery processes is a crucial step in replacing petrochemical products by sustainable, biotechnological alternatives. Substrate costs and downstream processing present large potential for improvement of cost efficiency. The implementation of in situ adsorption as an energy-efficient product recovery method can reduce costs in both areas. While selective product separation is possible at ambient conditions, yield-limiting effects, as for example product inhibition, can be reduced in an integrated process.

**Results:**

An in situ adsorption process was integrated into the production of itaconic acid with *Ustilago cynodontis* IA_max_, as an example of a promising biorefinery process. A suitable feed strategy was developed to enable efficient production and selective recovery of itaconic acid by maintaining optimal glucose concentrations. Online monitoring via Raman spectroscopy was implemented to enable a first process control and understand the interactions of metabolites with the adsorbent. In the final, integrated bioprocess, yield, titre, and space–time yield of the fermentation process were increased to values of 0.41 g_IA_/g_Glucose_, 126.5 g_IA_/L and 0.52 g_IA_/L/h. This corresponds to an increase of up to 30% in comparison to the first extended batch experiment without in situ product removal. Itaconic acid was recovered with a purity of at least 95% and high concentrations above 300 g/L in the eluate.

**Conclusion:**

Integration of product separation via adsorption into the bioprocess was successfully conducted and improved the efficiency of itaconic acid production. Raman spectroscopy was proven to be a reliable tool for online monitoring of various metabolites and facilitated design and validation of the complex separation and feed process. The general process concept can be transferred to the production of various similar bioproducts, expanding the tool kit for design of innovative biorefinery processes.

**Supplementary Information:**

The online version contains supplementary material available at 10.1186/s13068-023-02433-w.

## Background

Cost-efficient biorefinery concepts play a key role in the transition from a fossil-based economy to a circular bioeconomy. In the competition with petrochemical production processes, biochemical synthesis of platform chemicals is under immense cost pressure [1]. Major cost drivers of biorefinery processes include substrate costs and downstream processing [2, 3]. While substrate costs can be reduced by selecting cheap side streams, for example from agriculture and food processing, and by improving yields, the design of novel downstream processing concepts has been a popular research topic in recent years [4–8]. In contrast to conventional recovery and purification methods, as for example crystallisation and rectification, adsorption processes are energy-efficient, since they can usually be performed at ambient conditions [9–11]. Moreover, adsorption can be applied in situ [8, 12], allowing continuous product removal and reducing yield-limiting effects like product inhibition [5]. Possible concerns regarding bioprocess compatibility of activated carbons and nutrient adsorption have been investigated in Pastoors et al. [13] and no negative impact on the bioprocess was observed for IA production with *U. cynodontis*.

Different *Ustilago* strains have been shown to metabolise various complex substrates [14] and produce many interesting products, including biosurfactants [15] or intercellular lipids [16]. Meanwhile, the production of itaconic acid (IA) with Ustilaginaceae from renewable resources has received interest as a potential biorefinery process [17, 18]. Especially fermentations with engineered *U. maydis* and *U. cynodontis* strains reach high calculated titres, productivities and yields of up to 206 g_IA_/L, 0.59 g_IA_/L/h and 0.66 g_IA_/g_Glucose_ [19, 20], which is close to the theoretical yield of IA production from glucose of 0.72 g_IA_/g_Glucose_. However, the presented values for titre and yield were achieved in fermentations at neutral pH values, at which a recovery of IA with conventional downstream methods is connected to high costs and high loads of waste salts, because of the necessary acidification during subsequent downstream processing. For the experiment with a calculated titre of 206 g_IA_/L by Becker et al., this is even more of a problem, because the produced IA is precipitated as calcium itaconate and needs to be dissolved with equimolar amounts of HCl or H_2_SO_4_ before further purification. The space–time yield of 0.59 g_IA_/L/h was achieved in a process with significantly reduced yield of 0.3 g_IA_/g_Glucose_. In fermentations at acidic pH values, which are necessary for an efficient product recovery, IA production is hampered by product inhibition, caused by weak acid uncoupling [19, 21]. At acidic pH values and increased concentrations of organic acids, the protonated form of the acid can cross the cell membrane and enter the cell, decreasing the intracellular pH value. To maintain a stable pH in the cell, the acid has to be transported out of the cell, demanding increasing amounts of energy with increasing concentration of the acid outside of the cell. As a result, less of the carbon source can be used for product formation. In the remaining course of the cultivation, this effect leads to continuously reduced rates of product formation, until no more IA can be formed at concentrations of around 80 g/L [19, 21]. This effect has also been described for other organic acids like acetate, propionate and butyrate [22] and demands for in situ separation of the produced organic acid. Studies of Magalhaes et al. assessed adsorption as the most promising recovery method for biotechnologically produced IA [23, 24]. Adsorption on specific hydrophobic activated carbons has been shown to separate IA from glucose, displaying high adsorption capacities of 390 mg/g and separation factors of over 60 [25, 26].

This work focuses on integrating an in situ adsorption process with activated carbon into the production of IA with *U. cynodontis* at acidic pH values*.* It is the goal to demonstrate the potential of adsorption as an energy-efficient method for product recovery, without the formation of waste salts and improving key performance indicators (KPI) of the bioprocess, including yield, titre, and space–time yield. To control the different phases of product separation, a suitable technique for online monitoring of metabolite concentrations is necessary. As Raman spectroscopy has successfully been used for non-invasive monitoring of IA and glucose concentrations [27–30], Raman analysis is integrated into the setup to ensure optimal process control.

## Materials and methods

The materials and methods utilised in this work are similar to previous works [4, 13, 31].

The organism applied for IA production was *Ustilago cynodontis* NBRC9727 *Δfuz7*^*r*^* Δcyp3*^*r*^* P*_*etef*_*mttA P*_*ria1*_*ria1* (*U*. *cynodontis* IA_max_). *U*. *cynodontis* IA_max,_ was genetically engineered to grow in a yeast-like morphology and produce increased amounts of IA [19, 32]. The strain was kindly provided by Prof. Nick Wierckx (Institute of Bio- and Geosciences IBG-1: Biotechnology, Forschungszentrum Jülich, Jülich, Germany). For strain maintenance, stocks containing 200 g/L glycerol were used and stored at – 80 ℃.

### Media composition

For pre-cultivations of *U*. *cynodontis* IA_max_ an adapted Verduyn medium [33, 34] was used with the following concentrations: 50 g/L glucose, 4 g/L NH_4_Cl, 2 g/L KH_2_PO_4_, 0.4 g/L MgSO_4_ $\cdot$ 7H_2_O, 0.01 g/L FeSO_4_ $\cdot$ 7H_2_O, 19.52 g/L (0.1 M) MES-buffer and 1 mL/L trace element solution. The trace element solution contained 15 g/L EDTA, 3 g/L FeSO_4_ $\cdot$ 7H_2_O, 0.84 g/L MnCl_2_ $\cdot$ 2H_2_O, 4.5 g/L ZnSO_4_ $\cdot$ 7H_2_O, 0.3 g/L CuSO_4_ $\cdot$ 5H_2_O, 0.3 g/L CoCl_2_ $\cdot$ 6H_2_O, 0.4 g/L Na_2_MoO_4_ $\cdot$ 2H_2_O, 4.5 g/L CaCl_2_ $\cdot$ 2H_2_O, 1 g/L H_3_BO_3_ and 0.1 g/L KI. MgSO_4_ $\cdot$ 7H_2_O, NH_4_Cl and KH_2_PO_4_ were prepared separately, autoclaved, and stored at room temperature. The pH value of the KH_2_PO_4_ solution was adjusted to 6 with 10 M NaOH. All other media components were sterile filtered using a 0.2 μm cut-off cellulose acetate membrane filter (VWR International GmbH, Darmstadt, Germany). MES-buffer was adjusted to pH 6.5 with NaOH pellets. The trace elements solution and FeSO_4_ $\cdot$ 7H_2_O were stored at 4 ℃. For fermentations in the stirred tank reactor, an increased concentration of 100 g/L glucose and no MES-buffer was used. Instead, 10 M NaOH was used for pH control. Unless stated otherwise, all media components were diluted in deionised water (DI-water).

### Pre-cultivations in shake flasks

Pre-cultivations were performed in unbaffled 250 mL shake flasks using the Respiration Activity Monitoring System (RAMOS) [35, 36]. With the RAMOS device, the oxygen transfer rate (OTR), the carbon dioxide transfer rate (CTR) and the respiratory quotient (RQ) of each individual shake flasks were determined. Additionally, an ordinary Erlenmeyer flask was incubated under the same conditions. The cultivations were inoculated to an optical density value of 0.1 at a wavelength of 600 nm (OD_600_) from glycerol stock cell suspensions. The pre-culture was incubated at 30 ℃ with 10 mL initial filling volume, 350 rpm shaking frequency and 50 mm shaking diameter in a Climo-Shaker ISF1-X (Kühner AG, Birsfelden, Switzerland). Pre-cultures were harvested after around 30 h during exponential growth.

### Main cultivations in stirred tank reactors

Fermentation experiments were performed in a 2 L New Brunswick^™^ BioFlo^®^/CelliGen^®^ benchtop bioreactor (Eppendorf SE, Hamburg, Germany). The temperature was controlled at 30 ℃. The dissolved oxygen tension (DOT) was measured using a VisiFerm^™^ DO 225 pO_2_ sensor (Hamilton Bonaduz AG, Bonaduz, Switzerland) and maintained at 30% by variation of the agitation speed (300 rpm–800 rpm). A Rosemount X-Stream NGA 2000 exhaust gas analyser (Emerson Electric Co., St. Louis, USA) was used to determine the oxygen and carbon dioxide concentrations used for OTR, CTR and RQ calculations. The fermenter was equipped with two six-bladed Rushton turbines with a diameter of 51 mm and four baffles. The pH value was measured using an EasyFerm Plus K8 200 pH sensor (Hamilton Bonaduz AG, Bonaduz, Switzerland). At the beginning of each experiment and when needed to prevent foaming, 0.3 mL antifoam agent 204 (VWR International GmbH, Darmstadt, Germany) was added. Fermentations were started with an initial filling volume of 1.25 L, unless otherwise stated. The aeration rate was either set to 60 SL/h or adjusted depending on the filling volume to reach a mean volumetric aeration rate of q_in_ = 60 SL/L/h (1 vvm) depending on the experiment. The feed solution consisted of 500 g/L glucose and was sterile filtered. An Ismatec Reglo Analog ISM830 peristaltic pump (VWR International GmbH, Darmstadt, Germany) was used for the feed. The pH was controlled at 3.6 using 10 M NaOH with an initial pH value of 6. The volumes of the fed glucose solution and the titrated base solution were calculated based on gravimetric measurements and the densities, respectively. Volume change by NaOH titration, sampling, the glucose feed, and adsorption cycling was considered for mass balancing and all calculations. Fermentations were inoculated to an OD_600_ of 0.5 from the pre-culture.

### External loop setup and adsorption column

A detailed overview of the setup for in situ adsorption and desorption is given in the Results section of this work and in Additional file 1: Fig. S7. For cell retention, a PURON^**®**^ hollow fibre membrane (Koch Industries Inc., Wichita, USA) with a nominal pore size of 0.03 µm and a membrane area of 0.5 m^2^ was used. It was fixed in a spiral around all tubes and the DOT- and pH-probes, similar to the work of Carstensen et al. [37]. Two tubes were connected to the membrane ends with shrinking hoses as inlet and outlet of the inner membrane area. At the outlet, the tube was connected to the external loop.

For adsorption, SuperCompact 300–16, 300–26 or 600–16 adsorption columns (Götec Labortechnik GmbH, Bickenbach, Germany) were filled with a slurry of 96% v/v ethanol and Blücher 100562 activated carbon (Blücher GmbH, Erkrath, Germany). The total adsorbent volume in the packed columns amounted to 11.3 mL or 47 mL, depending on the experiment. Before the experiments, the ethanol was replaced with sterile DI-water to prevent ethanol introduction into the fermenter.

### Raman measurements

For Raman measurements, near-infrared immersion optics (0 mm focal distance) were connected to a 400 mW, 785 nm Raman analyzer (wavenumber range 160–3285 1/cm, 0.04 1/cm resolution, 3 × 5 s acquisition time, HoloGRAMS version 4.2, Endress+Hauser AG, Reinach, Switzerland) and built into airtight stainless steel flow cells (6 mm inner diameter T-piece, Swagelok Co., Solon, USA). For signal evaluation, PEAXACT software (version 5.7, S-PACT GmbH, Aachen, Germany) was used. Mass fractions of glucose, IA, erythritol and ethanol were calculated using indirect hard modelling [38–40]. For model calculations, a wavenumber range of 800–1800 1/cm was used. For transformation to volumetric concentrations, a correlation between the density of the fermentation supernatant and the concentrations of IA and glucose was established, based on density measurements in a DSA 48 density and sound analyzer (Anton Paar GmbH, Ostfildern-Scharnhausen, Germany). The correlation can be found in Additional file 1. Detailed information about the underlying models, the general Raman method and the measurement setup can be found in Additional files 1: Fig. SI 1, Table SI 1 and previous works [27, 28, 40]. Exemplary Raman spectra are displayed in Additional file 1: Figs. S2–S6.

### Offline analysis

The OD_600_ was photometrically measured at 600 nm in disposable semi-micro cuvettes (Brand GmbH & Co. KG, Wertheim, Germany) using a Genesys 20 spectrophotometer (Thermo Scientific Inc., Waltham, USA). Since a linear correlation for OD_600_ and cell mass was only viable for an OD_600_ between 0.1 and 0.3, samples were diluted using 9 g/L NaCl, if necessary. The pH was measured using a HI221 Basic pH-meter (Hanna Instruments Deutschland GmbH, Vöhringen, Germany), calibrated daily with two standard buffer solutions at pH 4 and 7.

For determination of the cell dry weight (CDW), 1 mL of fermentation broth was centrifuged in a Heraeus Multifuge X3R at 15,000 rpm for 5 min (Thermo Scientific Inc., Waltham, USA). The supernatant was discarded, and the cell pellet was washed with 1 mL of NaCl solution with a concentration of 9 g/L and centrifuged again. This procedure was repeated before the remaining pellet was dried at 70 ℃ for 48 h and weighed afterwards. Displayed CDW values are the mean of three technical replicates.

The determination of IA, glucose, erythritol and ethanol concentrations was carried out via high-performance liquid chromatography (HPLC). An Ultimate 3000 HPLC system (Thermo Scientific Inc., Waltham, USA) was used. The separating column was equipped with an Organic Acid Resin (300 × 7.8 mm, Phenomenex Ltd. Deutschland, Aschaffenburg, Germany), and an ERC RefractoMax 520 detector (IDEX Health & Science LLC, Kawaguchi, Japan). The flow rate of the mobile phase (5 mM H_2_SO_4_) was set to 0.8 mL/min with a column temperature of 60 ℃. Standards with concentrations between 0.064 g/L and 10 g/L were used to prepare the standard curves for all measurements. For HPLC measurement, fermentation samples were centrifuged for 3 min at 15,000 rpm. The supernatant was diluted with DI-water and sterile filtered with a Whatman^™^ 0.2-µm syringe filter (General Electric Co., Chicago, USA). For HPLC measurement of ethanol product solutions, ethanol was evaporated at room temperature and the resulting crystals were resuspended in DI-water and sterile filtered. The original concentration was then calculated based on the applied volumes of ethanol solution and DI-water.

### Calculations

For fermentations, the $OTR$ [mmol/L/h], $CTR$ [mmol/L/h] and $RQ$ [−] were calculated according to Eqs. (1–3), which can be derived from a stationary elemental balance around the headspace of the fermenter:

1 $$OTR= \frac{{q}_{In}}{{V}_{M}} \cdot \left({y}_{{O}_{2}, In}- \frac{1-{y}_{{O}_{2},In}-{y}_{{CO}_{2},In}}{1-{y}_{{O}_{2},Out}-{y}_{{CO}_{2},Out}} \cdot {y}_{{O}_{2},Out}\right),$$

2 $$CTR= \frac{{q}_{In}}{{V}_{M}} \cdot \left({y}_{{CO}_{2},Out}\cdot \frac{1-{y}_{{O}_{2},In}-{y}_{{CO}_{2},In}}{1-{y}_{{O}_{2},Out}-{y}_{{CO}_{2},Out}}- {y}_{{CO}_{2},In}\right),$$

3 $$RQ= \frac{CTR}{OTR}.$$

Here, ${q}_{In}$ [SL/L/h] is the volumetric aeration rate under standard conditions (25 ℃, 1 bar), ${V}_{M}$ is the molar gas volume with a value of 0.0224 SL/mmol, ${y}_{{O}_{2},In}$ [−] and ${y}_{{CO}_{2},In}$ [-] are the molar fractions of O_2_ and CO_2_ in the ingas and ${y}_{{O}_{2},Out}$ [−] and ${y}_{{CO}_{2},Out}$ [−] are the molar fractions of O_2_ and CO_2_ in the off-gas. The produced IA ${m}_{IA}$ [g] and consumed glucose ${m}_{Glucose}$ [g] were calculated according to Eqs. (4) and (5):

4 $${m}_{IA}= {c}_{IA}\cdot {V}_{L}+\sum_{{t}_{0}}^{t}{c}_{IA}\cdot {V}_{Sample}+\sum {c}_{IA,Waste}\cdot {V}_{Waste}+\sum {c}_{IA,Product}\cdot {V}_{Product}$$

5 $${m}_{Glucose}= {c}_{Glucose}\left({t}_{0}\right)\cdot {V}_{L}\left({t}_{0}\right)+ {c}_{Glucose,Feed}\cdot {V}_{Feed}- \sum_{{t}_{0}}^{t}{c}_{Glucose}\cdot {V}_{Sample}-\sum {c}_{Glucose,Waste}\cdot {V}_{Waste}-\sum {c}_{Glucose,Product}\cdot {V}_{Product} -{c}_{Glucose}\cdot {V}_{L}.$$

Here, ${c}_{IA}$ [g/L] is the concentration of IA and ${V}_{L}$ is the liquid volume in the fermenter. The term $\sum_{{t}_{0}}^{t}{c}_{IA}\cdot {V}_{Sample}$ represents the IA mass lost by sampling. The mass of IA in the waste tank after the washing steps (Fig. 3B) amounts to $\sum {c}_{IA,Waste}\cdot {V}_{Waste}$ and $\sum {c}_{IA,Product}\cdot {V}_{Product}$ represents the mass of IA collected in the product tank after the desorption steps (Fig. 3F). The term ${c}_{Glucose}\left({t}_{0}\right)\cdot {V}_{L}\left({t}_{0}\right)$ represents the initial glucose mass in the medium, while the glucose mass fed into the reactor in the extended batch phase is given by ${c}_{Glucose,Feed}\cdot {V}_{Feed}$. Similar to IA in Eq. (4), $\sum_{{t}_{0}}^{t}{c}_{Glucose}\cdot {V}_{Sample}$, $\sum {c}_{Glucose,Waste}\cdot {V}_{Waste}$ and $\sum {c}_{Glucose,Product}\cdot {V}_{Product}$ include glucose mass lost because of sampling, washing and desorption. As KPIs, the yield $Y$ [g_IA_/g_Glucose_] and space–time yield $STY$ [g_IA_/L/h] of the fermentations were calculated, based on ${m}_{IA}$ [g], ${m}_{Glucose}$ [g], the fermentation time $t$ [h] as well as the reaction volume ${V}_{Reaction, Max}$ of 2 L, according to Eqs. (6) and (7):

6 $$Y=\frac{{m}_{IA}}{{m}_{Glucose}},$$

7 $$STY=\frac{{m}_{IA}}{{V}_{Reaction, Max} \cdot t}.$$

## Results and discussion

### Adjustment of the feed strategy for constant and low glucose concentration

Low co-adsorption of glucose is a prerequisite for selective in situ separation of IA. Therefore, the glucose feed strategy for IA production in fermentations of *U*. *cynodontis* IA_max_ was adjusted in a first series of experiments. Glucose concentrations should be kept as low as possible throughout the bioprocess to reduce the concentration gradient driving the co-adsorption of glucose during in situ separation. At the same time, glucose concentrations around the glucose affinity constant K_S,Glucose_ should be avoided, to prevent limiting conditions and impaired growth and product formation [41]. Consequently, the aim was a constant glucose concentration around 5 g/L throughout the phases of product separation.

Therefore, the pulsed feed process, as described by Hosseinpour Tehrani et al. [19], was replaced by an extended batch process. This operation mode stands in contrast to a fed batch process, where glucose is fed at lower rates, resulting in limiting glucose concentrations [42]. This may be intended to avoid overflow metabolism, catabolite repression or excessive oxygen demand. In an extended batch process, however, the carbon source is constantly replenished to avoid limiting conditions, which would be unfavourable for IA production. In first extended batch processes with a linear feed, glucose accumulated at the end of the cultivation, indicating a reduced glucose uptake at later process stages. To account for the decreased glucose uptake, a fermentation with sequentially decreasing glucose feed rates was conducted (Additional file 1: Fig. S8).

As initial value, a feed rate of 4.1 g_Glucose_/h was calculated from the glucose uptake in a pulse experiment under similar conditions. The glucose feed was started with this feed rate after the initial batch phase at around 40 h. The feed rate was then reduced in three steps to a final value of 2.4 g_Glucose_/h, before the feed was stopped after 134 h. A similar course of respirational activity and IA production, as described by Hosseinpour Tehrani et al. [19], was observed, showing the general feasibility of the extended batch process. However, glucose accumulated during the feed phase up to concentrations of 95 g/L. Hence, glucose uptake was overestimated. To calculate the actual glucose uptake, the accumulation rate of glucose between the sampling points was subtracted from the respective feed rates. With this knowledge the glucose feed could be adjusted more precisely in the next experiment.

**Fig. 1:**
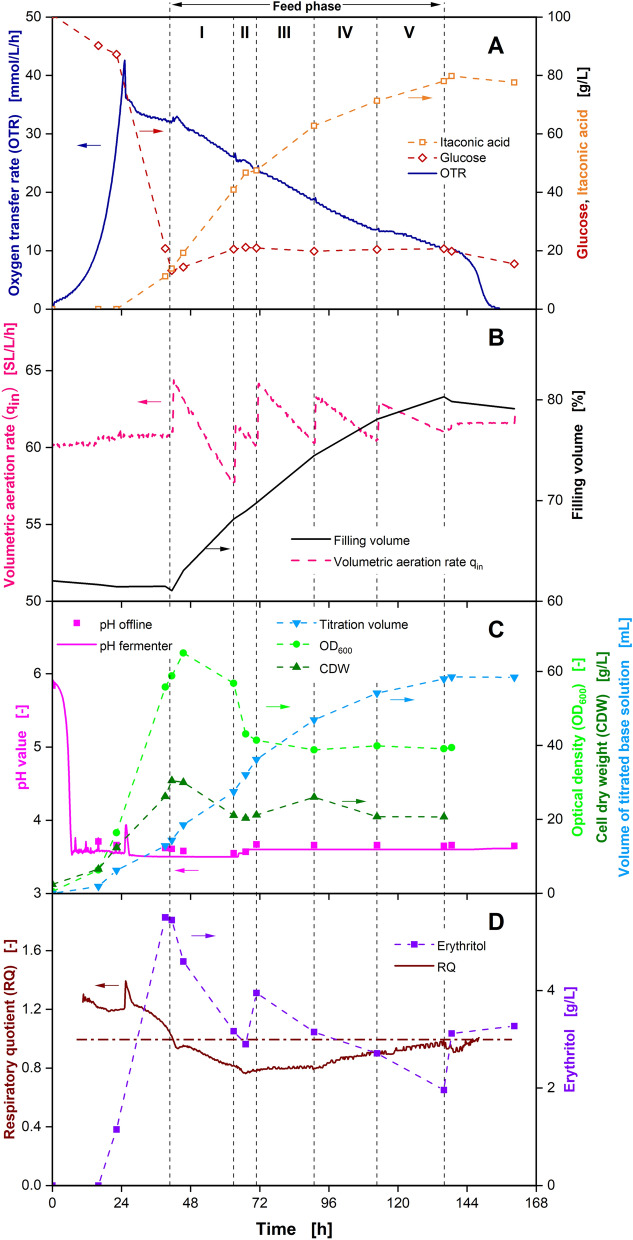
Extended batch cultivation of *U. cynodontis Δfuz7*^*r*^* Δcyp3*^*r*^* p*_*etef*_*mtta p*_*ria1*_*ria1* in a 2-L fermenter with 100 g/L initial glucose and sequentially decreasing glucose feed rates. Depicted are **A** oxygen transfer rate (OTR) and concentrations of glucose and itaconic acid, **B** volumetric aeration rate and filling volume, **C** pH, optical density (OD_600_), cell dry weight (CDW) and volume of titrated base solution and **D** respiratory quotient (line at RQ = 1) and erythritol concentration over time. Cultivation was performed at *T* = 30 ℃, *n* = 300–800 rpm, pH_initial_ = 5.92, pH_control_ = 3.6 (10 M NaOH), OD_600,initial_ = 0.5 and V_L,initial_ = 1250 mL in adapted Verduyn medium in a 2-L fermenter. Aeration rate adjusted at beginning of each feed phase to reach a mean volumetric aeration rate of q_in_ = 60 SL/L/h. Glucose was fed from a 517.3 g/L solution, resulting in feed rates of I: 3.6 g/h, II: 3.0 g/h, III: 2.4 g/h, IV: 1.9 g/h and *V*: 1.3 g/h. Feed rates were calculated from glucose accumulation in the experiment shown in Fig. SI 7. For concentrations, mean values of two replicates are shown

Based on the actual glucose uptake rates and the possible volumetric feed rates defined by the pump setup, an extended batch process with five feed phases was designed. The results of this extended batch fermentation are displayed in Fig. 1. The feed is started at 42 h with an initial feed rate of 3.6 g_Glucose_/h, which is decreased to 1.3 g_Glucose_/h over the course of 72 h and stopped after 134 h. During the first 24 h an exponential increase in OTR up to a maximum of around 45 mmol/L/h can be observed. At the same time, the glucose concentration starts decreasing, while the IA concentration remains at 0 g/L (Fig. 1A). OD_600_ as well as CDW also increase (Fig. 1C), indicating exponential growth in the first 24 h.

Meanwhile, the pH drops to 3.6 and pH control is initiated (Fig. 1C), which is connected to the uptake of NH_4_Cl for biomass formation. The RQ fluctuates around 1.2 during this period, indicating the formation of the reduced by-product erythritol, which is proven by the increase in erythritol concentration (Fig. 1D). Erythritol is known to be formed by *Ustilago* strains at elevated glucose concentrations and low pH values [19, 20].

After 24 h, a secondary substrate limitation is indicated by the peak and following decreasing plateau in the OTR. This can be identified as a nitrogen limitation, which is intentionally introduced for product formation in *Ustilago* cultivations [17, 19]. IA formation starts at the same time. In the following hours, glucose is converted into IA and biomass as evidenced by the course of the respective concentrations as well as OD_600_ and CDW. *Ustilago* strains are known to continue biomass production even after the introduction of a nitrogen limitation, which is facilitated by a change in biomass composition [21, 43]. The parallel formation of biomass and the oxidised product IA is further supported by the RQ, at values close to 1 during the first hours of nitrogen limitation.

To control the pH at the desired value of 3.6, a total of 60 mL NaOH is continuously fed into the reactor during IA production. After compensating the NH_4_Cl uptake in the batch phase, the volume of titrated base solution shows a good correlation to IA production (Fig. 1A–C). This presents a qualitative alternative for monitoring IA production, when no other online monitoring is available.

After 42 h, the glucose feed is started, with sequentially decreasing feed rates in five phases, increasing the filling volume in the reactor to around 80%. The aeration rate is adjusted at the start of each feed phase, to reach a mean volumetric aeration rate of 60 SL/L/h (Fig. 1B). As a result of the sequential feed rates, the glucose concentration remains nearly constant between 10 and 20 g/L until the end of the cultivation. Hence, the aim of reaching a reduced and constant glucose concentration is fulfilled by the adjusted feed strategy.

For the rest of the cultivation, IA production continues, reaching a final titre of around 80 g/L after 160 h. At 70 h and an IA concentration of 50 g/L the slope of the IA concentration decreases, indicating a decline in product formation, caused by product inhibition. This is due to the aforementioned weak acid uncoupling [19, 21]. Consequently, IA production is limited to a final titre of 80 g/L, with a yield of 0.38 g_IA_/g_Glucose_, although glucose is still available at the end of the process.

After the start of the feed phase no more biomass is formed, as the nitrogen content of the biomass has supposedly reached its minimum and no other nitrogen source is available. Meanwhile, erythritol is consumed by *U. cynodontis* IA_max_, leading to an overall decrease in erythritol concentration over the rest of the feed phase. As a result, the RQ drops to values below 1 for the rest of the cultivation. The erythritol metabolism of *U. cynodontis* IA_max_ is strongly dependent on the glucose concentration [19]. As repeated switches from formation to consumption of erythritol can be noticed in this experiment, the critical glucose concentration for erythritol metabolism is most likely between 10 and 20 g/L. Consequently, in an optimised production process, glucose concentration should be kept at lower values for minimal by-product formation.

### Developing an in situ adsorption setup for repeated itaconic acid recovery

After implementing the sequential feed strategy, the challenge of product inhibition by weak acid uncoupling remains. As discussed before, the most effective measure against product inhibition is the removal of the product from the fermentation broth [5]. In this work, in situ adsorption on an activated carbon was implemented to repeatedly reduce the concentration of IA in the fermenter, with the goal of ensuring non-inhibiting conditions.

**Fig. 2:**
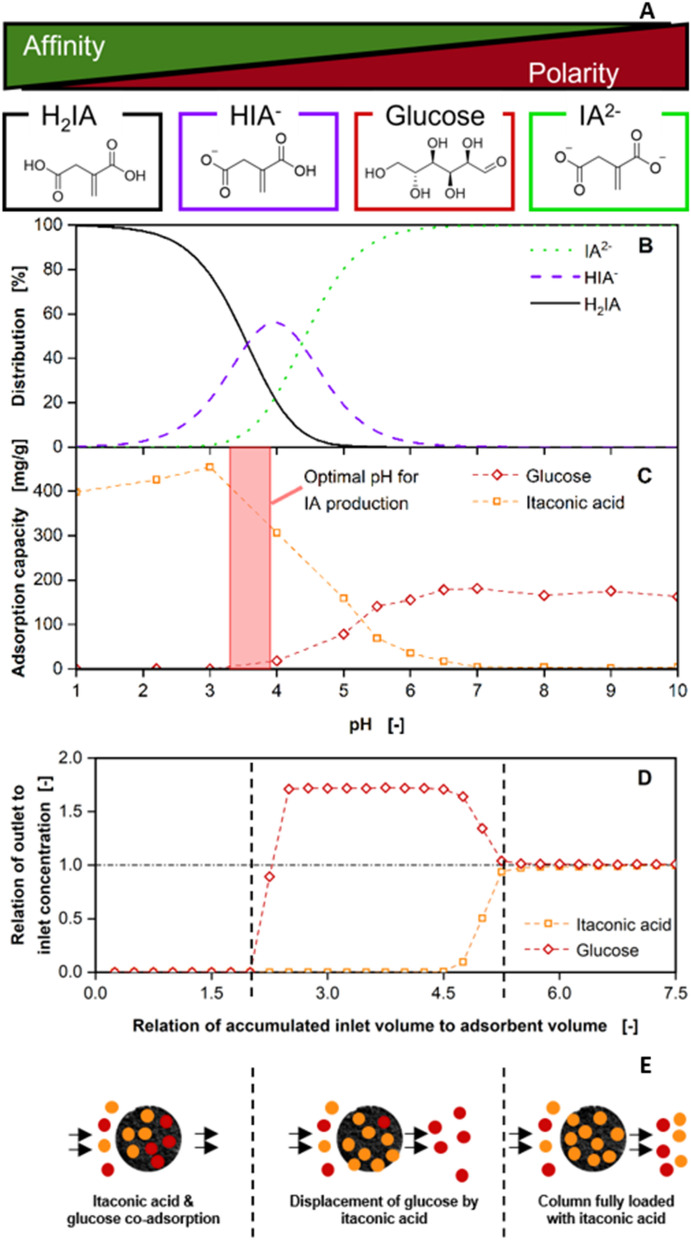
Adsorption characteristics for itaconic acid and glucose on activated carbons. **A** Relation between polarity and affinity to hydrophobic activated carbons for the different itaconic acid species and glucose. Metabolites depicted in order of decreasing affinity. H_2_IA: fully protonated itaconic acid, HIA^−^: single protonated itaconic acid, IA^2−^: fully dissociated itaconic acid. **B** pH-dependent distribution of itaconic acid species as described in [45]. **C** pH-dependent adsorption capacity of itaconic acid and glucose from a 1:1 mixture (30 g/L) on Blücher 100562 activated carbon at *T* = 30 ℃ after 1 h of batch adsorption. **D** Relation of outlet to inlet concentration for itaconic acid and glucose over the relation of accumulated inlet volume to adsorbent volume for a continuous adsorption on an adsorption column. The column was filled with 4 mL of activated carbon. The adsorption experiment was performed with a 1:1 mixture (60 g/L) at *T* = 30 ℃ and an inlet flow rate of V_F_ = 1 mL/min. **E** Illustration of the distribution of metabolites adsorbed to the column for the three different phases from the experiment depicted in **D**

Since the work of Pastoors et al. [13] showed no co-adsorption of other metabolites, the general adsorption characteristics of mixtures of IA and glucose on the activated carbon was studied. The affinity of a metabolite to hydrophobic activated carbons is strongly dependent on the polarity [44]. Since IA has two different dissociation states with varying polarity, different adsorption behaviours are to be expected, as already described for hydrophobic polymers by Biselli et al. [27]. As displayed in Fig. 2A, the fully protonated IA (H_2_IA) has the highest affinity towards the activated carbon in our system, followed by the single protonated species (HIA^−^), glucose and the fully dissociated species (IA^2−^). As the protonation state of IA is mainly dependent on the pH value (Fig. 2B), the pH of the fermentation is crucial for the success of IA separation.

When performing a batch adsorption with activated carbon and a mixture of IA and glucose, adsorption capacities are strongly dependent on the pH value, as displayed in Fig. 2C. While at acidic pH values almost only IA is recovered with adsorption capacities above 400 mg/g, the adsorption capacity shifts towards glucose with increasing pH values. This is caused by a shift in IA distribution to the more polar forms, which have a lower affinity to the activated carbon than H_2_IA. As a result, IA is adsorbed in lesser amounts, vacating adsorption sites that are then available for glucose adsorption. Consequently, the capacity for glucose adsorption increases. Above pH values of 7, exclusively glucose is separated with capacities of around 150 mg/g, since IA is completely present as the strongly polar form IA^2−^. However, at the optimal pH of 3.6 for IA production with *U. cynodontis* IA_max_, IA is mainly present as H_2_IA. Therefore, IA adsorption is favourable at fermentation conditions.

Since adsorptive IA recovery is integrated into the bioprocess in a continuous mode of operation, continuous separation of IA–glucose mixtures was investigated, as displayed in Fig. 2D. A 1:1 mixture (*w*/*w*) was fed into an adsorption column filled with 4 mL of activated carbon with a constant flow rate of 1 mL/min, while concentrations of IA and glucose at the outlet of the column were monitored. The relation of outlet to inlet concentration of IA and glucose is displayed over the accumulated inlet volume relative to the bed volume of activated carbon in the column.

No IA or glucose is detected at the end of the column until the accumulated inlet volume reaches two column volumes. Then, the relation of outlet to inlet concentration for glucose increases to 1.8. After the accumulated inlet volume equals five bed volumes, IA concentration at the outlet of the column increases to the value of the inlet concentration, while at the same time glucose concentration decreases to the inlet concentration. This adsorption process can be divided into three phases, as indicated by the vertical dashed lines illustrated in Fig. 2E. During the first phase, co-adsorption of IA and glucose takes place until all adsorption sites are occupied. As a result, no IA or glucose leave the column. When the column is fully loaded, adsorbed glucose is displaced by IA, because of its higher affinity to the activated carbon in the second phase. This results in an increased glucose concentration at the column outlet, compared to the inlet. When the column is fully loaded with IA at the start of the third phase, all fed IA and glucose pass the column and the mixture reaches the end of the column with inlet concentrations. Consequently, very low amounts of glucose remain on the column after continuous adsorption. Therefore, the selectivity for IA recovery against glucose on activated carbons is further increased in comparison to the batch adsorption displayed in Fig. 2B, highlighting the potential of adsorption for in situ recovery of IA.

In a next step, the setup for in situ recovery had to be designed. As columns filled with activated carbon are densely packed, cells would clog the column and lead to high pressure drops. Consequently, a measure for cell retention in the fermenter is necessary. To supply the external loop with cell-free fermentation supernatant, a hollow fibre membrane was built into the fermenter.

**Fig. 3:**
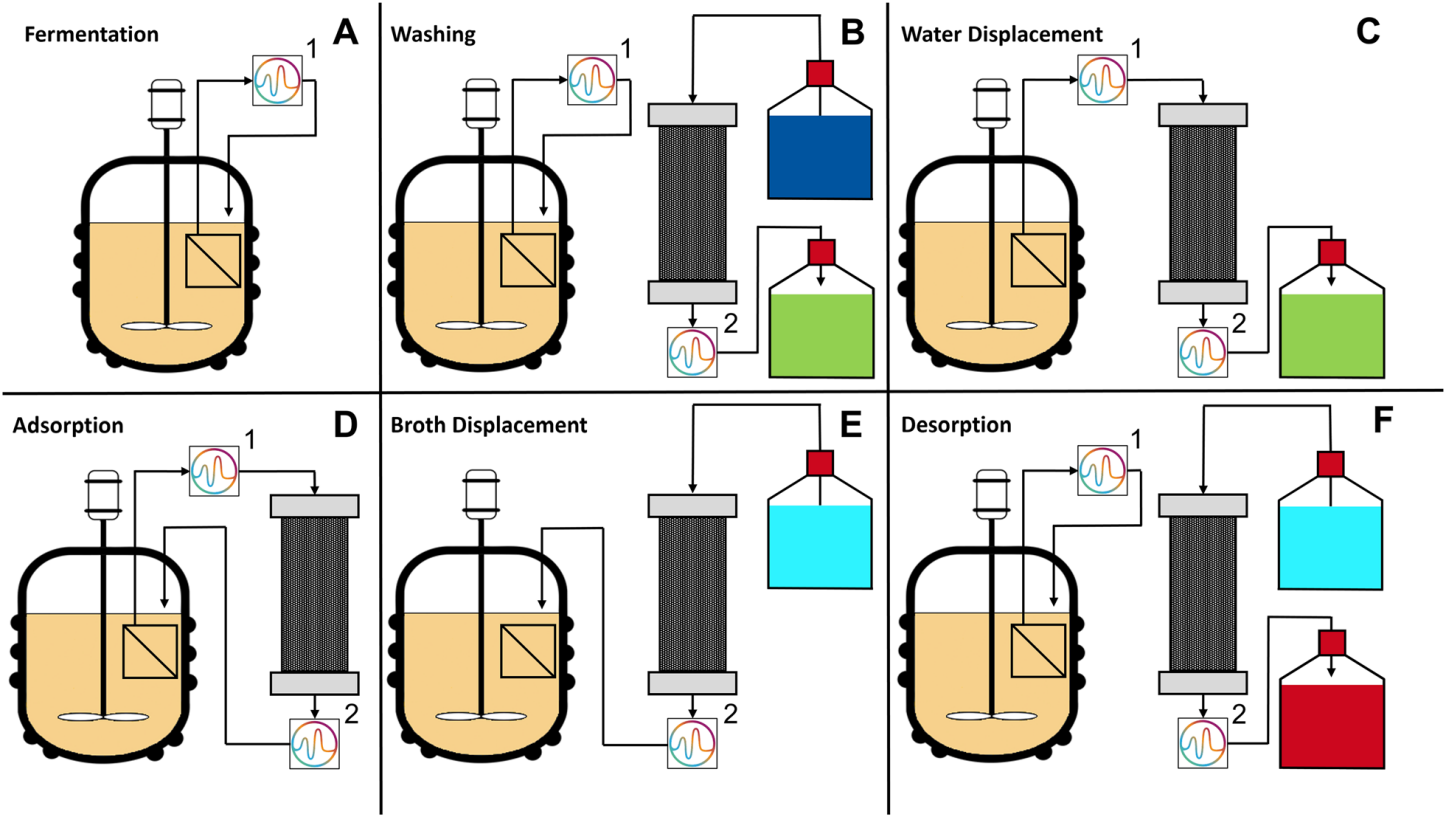
Simplified illustration of liquid flows for in situ adsorption and Raman analysis for the different phases of product separation cycles. A schematic drawing of the complete experimental setup with all pumps and valves is shown in Additional file 1: Figure S7. Tanks are filled with DI-water (dark blue), waste (green), ethanol (light blue) and product solution (red). Flow directions during different steps: **A** fermentation: bioreactor → Raman sensor 1 → bioreactor. **B** Washing: water tank → adsorption column → Raman sensor 2 → waste tank (fermentation step is run simultaneously). **C** Water displacement: bioreactor → Raman sensor 1 → adsorption column → Raman sensor 2 → waste tank. **D** Adsorption: bioreactor → Raman sensor 1 → adsorption column → Raman sensor 2 → bioreactor. **E** Broth displacement: ethanol tank → adsorption column → Raman sensor 2 → bioreactor. **F** Desorption: ethanol tank → adsorption column → Raman sensor 2 → product tank (fermentation step is run simultaneously)

A detailed overview of the complete experimental setup with all pumps and valves is shown in Additional file 1: Fig. S7. By applying a pressure difference with pumps connected to the inside of the membrane, outside-in filtration was performed, retaining the cells outside of the membrane in the fermenter. To monitor the concentrations of glucose and the different IA species, two flow cells for Raman measurement were integrated into the setup. During the IA production process, the flow directions in the setup were adjusted depending on the phases of product separation, as displayed in Fig. 3.

During regular fermentation (Fig. 3A), the supernatant was pumped through the first Raman cell for monitoring of concentrations in the fermenter. To clear the column of ethanol used for sterilisation or desorption, sterile DI-water was pumped during the washing step (Fig. 3B) from the water tank (dark blue) over the column and through the second Raman flow cell into the waste tank (green). Thereby, the concentrations of glucose, ethanol and the different IA species at the column outlet can be monitored. Simultaneously, the fermentation step (Fig. 3A) was kept running, to maintain Raman analysis in the supernatant. Before performing an adsorption step, the water remaining in the column from washing was displaced with supernatant from the fermenter and flushed into the waste tank (Fig. 3C). When the supernatant reached the bottom of the column, monitored by Raman spectroscopy, the outflow of the column was redirected back to the fermenter for the adsorption step (Fig. 3D). In that way, the remaining nutrients were kept available for the cultivation and the medium in the fermenter was refilled with the IA-depleted supernatant. When the column was fully loaded, the supernatant remaining in the column was displaced with ethanol (light blue) for desorption and pumped back into the fermenter (Fig. 3E). When ethanol reached the outflow of the column, the flow was redirected to the product tank for the desorption step (Fig. 3F, red). While running the fermentation step for Raman analysis in parallel, desorption was continued until the column was depleted and the next washing step (Fig. 3B) was performed.

### Integrating in situ adsorption into the bioprocess for itaconic acid production

**Fig. 4:**
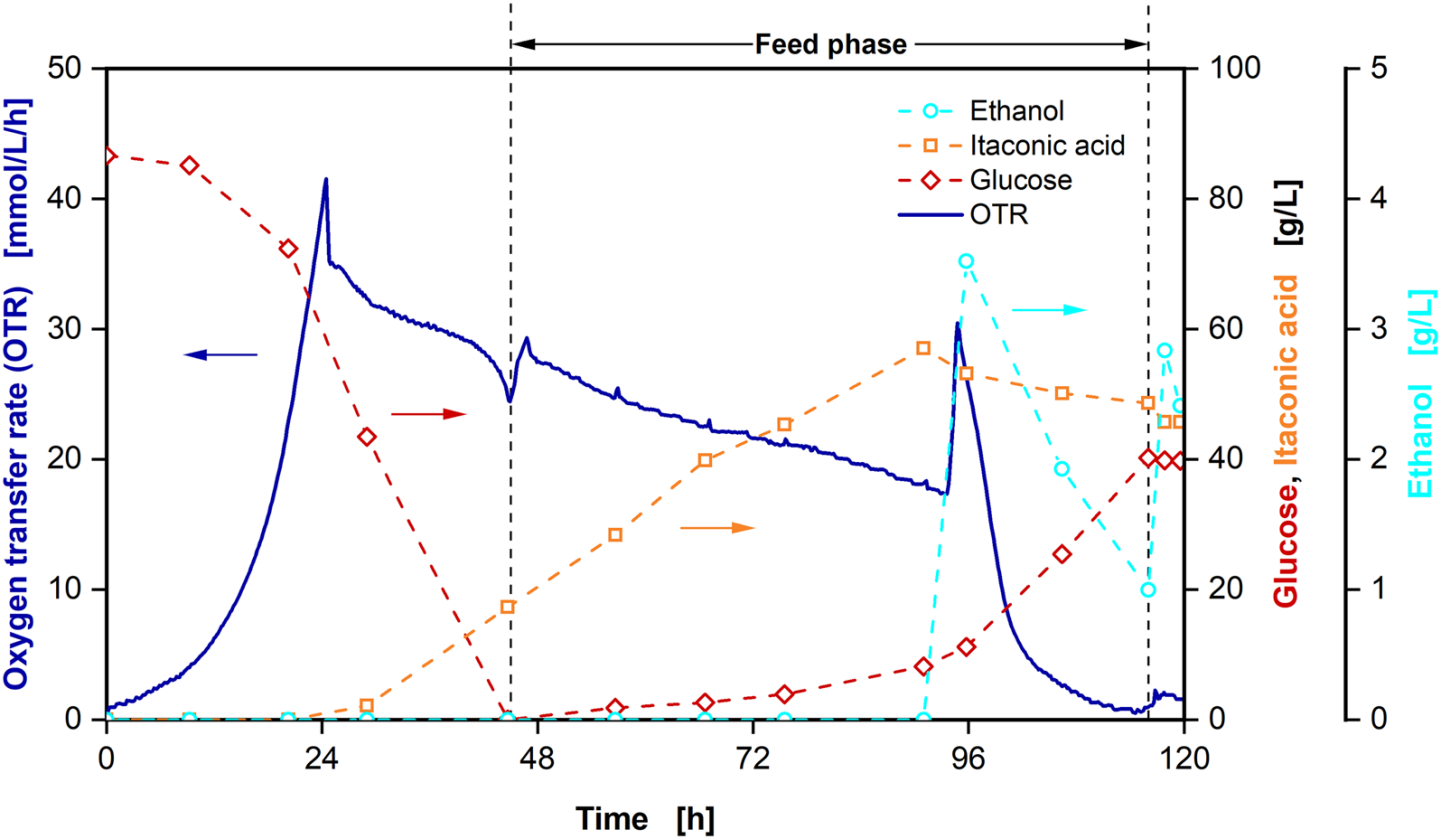
Proof of concept cultivation of *U. cynodontis Δfuz7*^*r*^* Δcyp3*^*r*^* p*_*etef*_*mtta p*_*ria1*_*ria1* in a 2 L fermenter with 100 g/L initial glucose, a linear glucose feed rate and in situ separation of itaconic acid by adsorption. Depicted are oxygen transfer rate (OTR) and concentrations of glucose, itaconic acid and ethanol over time. Cultivation was performed at *T* = 30 ℃, *n* = 300–650 rpm, q_in_ = 60 SL/L/h, pH_initial_ = 5.78, pH_control_ = 3.6 (10 M NaOH), OD_600,initial_ = 0.5 and V_L,initial_ = 1250 mL in adapted Verduyn medium in a 2-L fermenter. DOT was controlled at 30% by adjusting stirrer speed. Glucose was fed from a 440.7 g/L solution. Column was filled with 11.3 mL of Blücher 100562 activated carbon. The adsorption steps of product separation cycles were performed from 92 to 96 h and 116–118 h. For concentrations, mean values of two replicates are shown

As a first proof of concept, the in situ adsorption process was conducted with an adsorbent volume of 11.3 mL in the column. To keep process control as simple as possible, a linear glucose feed was applied. Otherwise, the same conditions were applied for the bioprocess as for the fermentation presented in Fig. 1. The Raman device was not available for this experiment, so the durations of the steps of the product separation cycles had to be calculated from an assumed product concentration in the bioreactor as well as the knowledge of the capacity of the utilised adsorbent. The results of the experiment are displayed in Fig. 4.

The unlimited part of the batch phase until 24 h indicates the same trends for OTR and concentrations of IA and glucose, as described for Fig. 1. At the end of the batch phase after 46 h, the OTR drops shortly, indicating glucose depletion before the feed is started. Until the first product separation cycle, IA is produced, and glucose is accumulated in amounts of < 10 g/L. After 92 h, the adsorption step of the first product separation cycle was started. As a result, the concentration of IA in the reactor is reduced by around 5 g/L through dilution with IA-depleted supernatant flowing back from the adsorption column to the fermenter.

However, unintentionally ethanol is simultaneously introduced into the fermenter, reaching a concentration of around 4 g/L. Since no monitoring of concentrations at the outflow of the column was available, process control was not optimal, and the washing step was not run long enough. Thus, ethanol from the sterilisation remained in the column and was flushed into the fermentation, demonstrating the need for an online monitoring of concentrations at the outlet of the column by Raman spectroscopy. Ethanol is known to be toxic for various microorganisms at elevated concentrations [46–48]. Combined with the difficulties connected to the nitrogen limitation and weak acid stress, all metabolic activity of *U cynodontis* IA_max_ ceased, indicated by a sharp drop in OTR after 93 h. Glucose was no longer converted into IA, resulting in an accumulation of glucose from the feed. IA concentrations showed a slight decline over the next few hours, which is caused by dilution by the glucose feed. After the OTR reached values around 0 mmol/L/h, the feed was stopped, and the process was terminated. Consequently, introduction of elevated amounts of ethanol into the fermenter has to be prevented by all means when performing in situ adsorption.

**Fig. 5:**
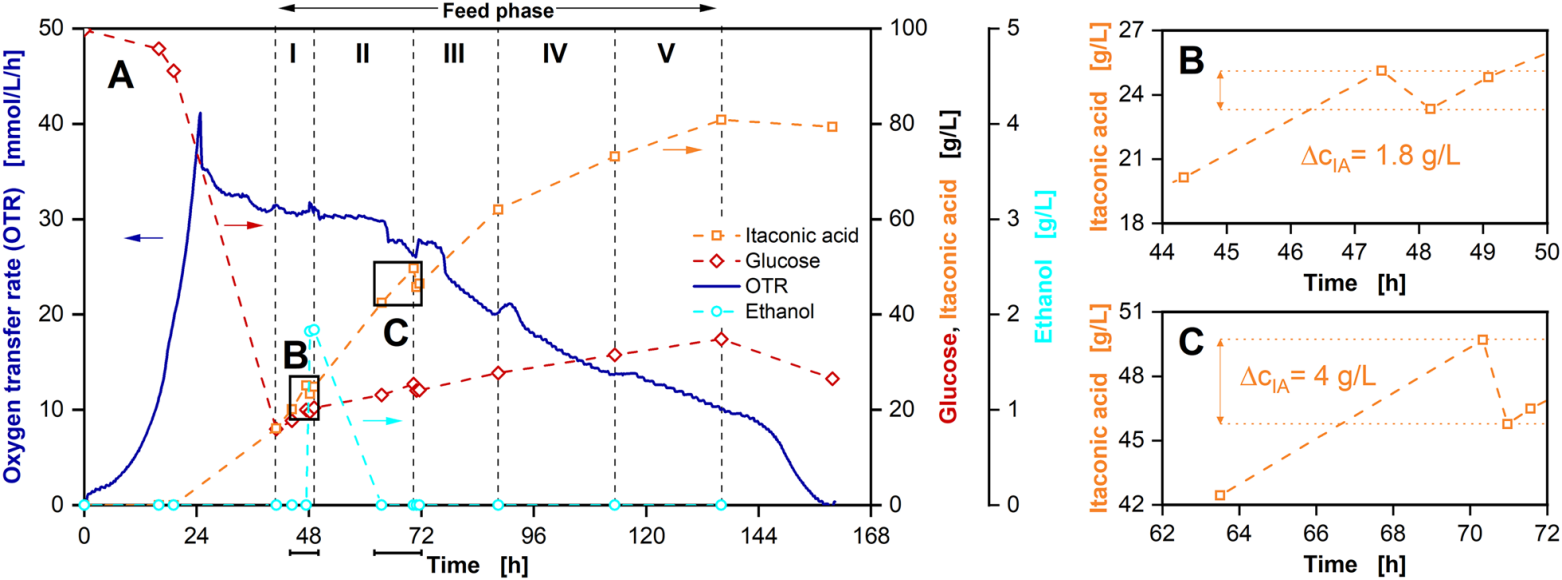
Proof of concept cultivation of *U. cynodontis Δfuz7*^*r*^* Δcyp3*^*r*^* p*_*etef*_*mtta p*_*ria1*_*ria1* in a 2 L fermenter with 100 g/L initial glucose, sequentially decreasing glucose feed rates and in situ separation of itaconic acid by adsorption. Depicted are **A** oxygen transfer rate (OTR) and concentrations of glucose, itaconic acid and ethanol over time. **B**, **C** Are enlarged representations of concentrations of itaconic acid during adsorption cycles over time. Cultivation was performed at *T* = 30 ℃, *n* = 300–600 rpm, q_in_ = 60 SL/L/h, pH_initial_ = 5.75, pH_control_ = 3.6 (10 M NaOH), OD_600,initial_ = 0.5 and V_L,initial_ = 1250 mL in adapted Verduyn medium in a 2-L fermenter. DOT was controlled at 30% by adjusting stirrer speed. Glucose was fed from a 500.3 g/L solution, resulting in feed rates of I: 4.0 g/h, II: 3.3 g/h, III: 2.6 g/h, IV: 1.9 g/h and V: 1.2 g/h. Column was filled with 11.3 mL of Blücher 100562 activated carbon. The adsorption steps of product separation cycles were performed from 47.7 to 48.1 h and 70.6 to 70.9 h. For concentrations, mean values of two replicates are shown

A second proof of concept experiment was conducted with a doubled duration of the washing step and the sequentially decreasing feed profile established in the experiment displayed in Fig. 1. The results are shown in Fig. 5.

Again, OTR and concentrations of IA and glucose show the same trends during the batch phase, with a peak after 24 h at around 42 mmol/L/h, underlining the reproducibility of the presented bioprocess. Shortly after the start of the feed phase at 47.7 h, the first product separation cycle was initiated. During adsorption, the concentration of IA in the fermenter was reduced by around 1.8 g/L, as visible in Fig. 5B. Although washing time before the cycle was doubled, ethanol was introduced into the fermenter together with the IA-depleted supernatant, resulting in an ethanol concentration of 2 g/L. However, no negative impact on *U. cynodontis* IA_max_ was observed this time. Instead, ethanol concentration dropped to 0 g/L, being either consumed by *U. cynodontis* IA_max_ or evaporated over time.

Following the product separation cycle, IA production continues until the next separation cycle is started at 70.6 h. This time, IA concentration in the fermenter decreased by 4 g/L, as displayed in Fig. 5C. Afterwards, IA is produced until the same titre of 80 g/L is reached, as in the experiment without in situ adsorption. Consequently, the effect of product inhibition could be delayed by 2–3 h. The total IA titre was increased through in situ adsorption by around 4.8 g/L, when related to the final volume of the experiment and compared to the process displayed in Fig. 1. However, the amount of separated IA was limited by the volume of adsorbent in the column. Moreover, glucose was again accumulated during the feed phase and not completely metabolised at the end of the experiment, leaving room for further improvement of the process.

### Final process with in situ adsorption on larger column and concentration monitoring via Raman

**Fig. 6:**
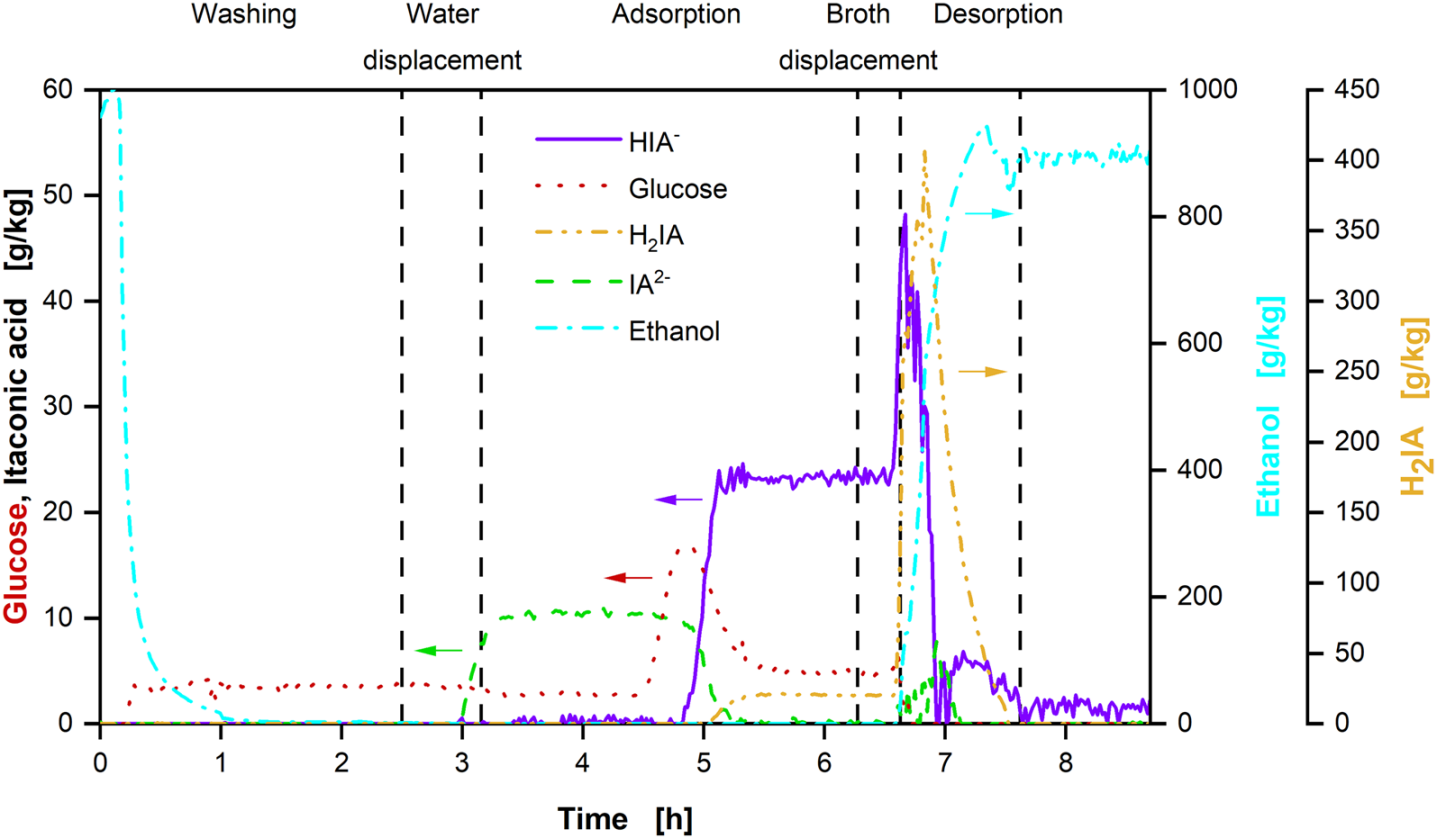
Mass fractions of different metabolites calculated from the Raman signal of flow cell 2 (see Additional file 1: Fig. S7) behind the adsorption column. Depicted are mass fractions of the analysed Raman-active substances over time and the different phases of the product separation cycle according to Fig. 3. Start and end points of different phases in the experiment were adjusted according to the measured Raman signals. The displayed product separation cycle is the second cycle from the experiment displayed in Fig. 7 (87.4–99.5 h). The column was filled with 47 mL of Blücher 100562 activated carbon

An experiment was performed with a larger adsorption column, resulting in a total adsorbent volume of 47 mL and, thereby, a more than threefold increase in total adsorption capacity. Moreover, Raman monitoring was integrated to improve the feed strategy and control of cycle times. The concentrations calculated from the Raman signals of a complete product separation cycle from flow cell 2 (Additional file 1: Fig. S7) behind the column are displayed in Fig. 6.

In the first 2.5 h, ethanol is flushed out of the column during the washing step (Fig. 3B). When no more ethanol leaves the column, water displacement is started (Fig. 3C). The first metabolite from the supernatant reaching the outlet of the column is the strongly polar IA^2−^. It has a low affinity to the hydrophobic activated carbon (Fig. 2) and, therefore, passes the column faster than the other metabolites. As soon as it is detected, the flow behind the column is redirected to the fermenter and the adsorption step is started (Fig. 3D).

The remaining metabolites leave the column in the order of increasing affinity to the adsorbent, as soon as it is fully loaded. First, glucose concentration shows a sharp increase at 4.5 h, before dropping to a constant plateau at around 5 g/kg, which is caused by the displacement effect described in Fig. 2E. Glucose concentrations before 4.5 h are probably overestimated, because of inaccuracies in the model for evaluation of the Raman signal at low glucose concentrations. No glucose is to be expected at column outflow at that time. Then, HIA^−^ concentration increases after 4.8 h, until a plateau is reached at around 23 g/kg. As the last metabolite, H_2_IA leaves the column after 5 h, increasing to a constant concentration of around 21 g/kg. When all metabolites reached a constant level after around 6.3 h, ethanol was fed into the column for broth displacement (Fig. 3E). After around 6.6 h, ethanol is detected and the flow from the column is redirected to the product tank (Fig. 3F). During desorption, high concentrations of 400 g/kg and 50 g/kg for H_2_IA and HIA^−^ are measured in the desorbate, facilitating further purification. Meanwhile, no glucose and only low amounts of IA^2−^ are desorbed from the column. When no more IA is detected in the desorbate, the desorption is terminated.

**Fig. 7:**
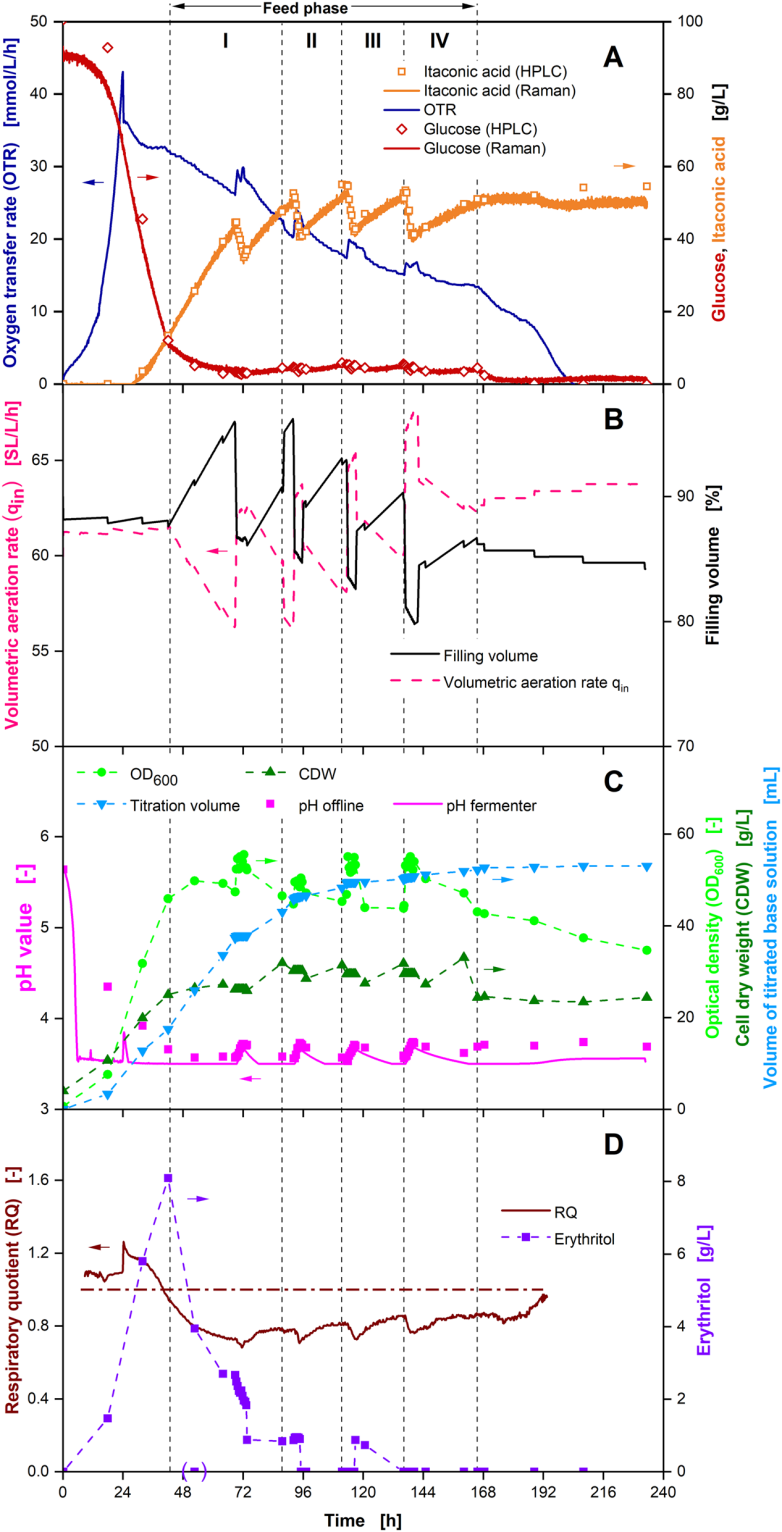
Cultivation of *U. cynodontis Δfuz7*^*r*^* Δcyp3*^*r*^* p*_*etef*_*mtta p*_*ria1*_*ria1* in a 2-L fermenter with 100 g/L initial glucose, sequentially decreasing glucose feed, in situ separation of itaconic acid by adsorption and concentration monitoring by Raman spectroscopy. Depicted are **A** oxygen transfer rate (OTR) and concentrations of glucose and itaconic acid, **B** volumetric aeration rate and filling volume, **C** pH, optical density (OD_600_), cell dry weight (CDW) and volume of titrated base solution and **D** respiratory quotient (line at RQ = 1) and erythritol concentration over time. Cultivation was performed at *T* = 30 ℃, n = 300–800 rpm, Q_in_ = 60 SL/h, pH_initial_ = 5.67, pH_control_ = 3.6 (10 M NaOH), OD_600,initial_ = 0.5 and V_L,initial_ = 1800 mL in adapted Verduyn medium in a 2 L fermenter. DOT was controlled at 30% by adjusting stirrer speed. Glucose was fed from a 510.2 g/L solution resulting in feed rates of I: 3.4 g/h, II: 2.8 g/h, III: 2.0 g/h and IV: 1.6 g/h. Column was filled with 47 mL of Blücher 100562 activated carbon. The adsorption steps of product separation cycles were performed from 68.9 to 73.3 h, 92.2 to 95.7 h, 113.4 to 116.9 h and 136.4 to 140.4 h. For concentrations from HPLC measurements, mean values of two replicates are shown

The presented product separation procedure with Raman monitoring was incorporated into the IA production bioprocess (Fig. 7)*.*

The adsorption steps of four product separation cycles are clearly visible in the IA concentration during the production phase (68.9–73.3 h, 92.2–95.7 h, 113.4–116.9 h and 136.4–140.4 h), yielding to a decrease of IA concentration in the bioreactor of 9.6 g/L, 11.8 g/L, 12.8 g/L and 12.1 g/L of IA, respectively (Fig. 7A). As a result, IA concentration remains below 50 g/L for the majority of the process, reducing the influence of product inhibition on the cells. The dilution of the fermentation broth with IA-depleted supernatant not only leads to a drop in IA concentration, but also results in an increase in pH during adsorption (Fig. 7C). The pH of the supernatant increases by removal of IA in the adsorption column. The less acidic supernatant is then redirected to the fermenter, leading to the observed pH increase. Consequently, the amount of base used for titration can be reduced by application of in situ adsorption, further improving the potential for cost reduction of this technology.

Small peaks in the OTR can be observed at the start of each product separation cycle (Fig. 7A). The peaks are caused by the temporary volume loss connected to filling the loop with supernatant (Fig. 7B). While the volume in the reactor is reduced during water displacement after around 72 h, the total amount of cells remains the same, resulting in increased CDW and OD_600_ (Fig. 7C). At the same time, the volumetric aeration rate is increased by the volume loss, resulting in the observed peaks in the OTR. The first product separation cycle was paused after adsorption. Desorption was initiated in the next morning after around 90 h, visible in a rapid increase of filling volume during broth displacement (Fig. 7B). As a result, OD_600_, CDW and q_in_ decrease again. No negative influence of the delayed desorption step on the bioprocess is visible. Consequently, product separation and further purification are timely independent of each other.

The same trends in OTR, OD_600_ and CDW can be observed for the following product separation cycles. Meanwhile, IA and glucose concentrations calculated from the Raman signals are in excellent agreement with concentrations quantified by HPLC analysis for the whole process (Fig. 7A). Only minor deviations can be observed at very high glucose concentrations at the start of the process and for IA concentrations in the last 24 h. Glucose, which is fully consumed at the end of the process, is overestimated in the Raman predictions, while IA predictions represent lower values than HPLC results. At the edge of the calculation range for Raman predictions, model constraints possibly lead to a false identification of parts of the peaks in the Raman spectrum. During the feed phase, glucose predictions from the Raman analysis were used for the adjustment of feed rates.

As a result, glucose concentration was successfully kept constant at a desired value around 5 g/L for the whole feed phase. Consequently, erythritol formation in the bioprocess and co-adsorption of glucose during IA separation were reduced. While erythritol is produced during the batch phase (Fig. 7D) in similar amounts as displayed in Fig. 1, erythritol concentration remains below 2 g/L after 72 h. Moreover, a complete consumption of the remaining glucose at the end of the process is guided by the Raman monitoring.

In addition to the fermentation broth, the ethanol solution from the product tank was analysed via HPLC to quantify the separated IA. An exemplary chromatogram of the product solution from the desorption step of the second product separation cycle after evaporation, resuspension and dilution is displayed in Additional file 1: Fig. S9. Except for the IA peak, which can be quantified to a concentration of 6.3 g/L in the sample, only small peaks are visible in the chromatogram. For glucose, a concentration of 0.02 g/L is calculated, while erythritol is below the detection limit. Since the correlation between peak area and concentration differs for different substances, no exact concentrations can be calculated for the remaining, unknown substances. However, peaks can be compared to estimate the order of magnitude of the respective concentrations. When relating the glucose concentration to the IA concentration, it can be quantified as an impurity of 0.32% *w*/*w*. The remaining four distinct peaks at retention times of 6, 8, 13 and 18 min should amount to similar weight ratios. Consequently, a conservative estimation of the purity of the separated IA of above 95% *w*/*w* is calculated. As a result, the recovered IA can be used after limited further purification.

The KPIs of the experiments presented in this work are summarised in Table 1. When comparing titres, it is clearly visible that titres are increased, when in situ adsorption was successfully applied (Experiments 3 and 4). Separating IA during the process allows for more IA to be produced, preventing the product inhibition at IA titres around 80 g/L. This effect is increased with higher adsorption capacities. In addition, space–time yields are improved by application of in situ adsorption in a similar way. The duration of the growth phase is constant over the experiments, while the production phase is prolonged by IA separation by up to 60 h, increasing space–time yields by up to 11% to a value of 0.52 g_IA_/L/h.

**Table 1:** Overview of experiments and resulting KPIs including IA separated by in situ adsorption, following Eqs. (4–7)

Experiment	Figure	Titre [g_IA_/L]	Space–time yield [g_IA_/L/h]	Yield [g_IA_/g_Glucose_]	Number of product separation cycles [−]
1	1	77.6	0.47	0.38	0
2	4	51.8	0.42	0.3	2
3	5	84.4	0.49	0.41	2
4	7	126.5	0.52	0.41	4

Final titres are calculated and include IA in the fermenter as well as IA separated by in situ adsorption related to the final liquid volume in the fermenter

At the same time, yields for IA from glucose are also increased by in situ adsorption. While the amount of biomass is defined by the supplied nitrogen amount and remains constant over the experiments in this work, more glucose can be converted into IA, when IA is separated during production. The ratio of product to biomass is shifted, increasing the yield. However, yields in experiments 3 and 4 are very similar at values around 0.4 g_IA_/g_Glucose_. This is caused by a decreasing productivity of the cells with increasing duration of the nitrogen limitation. The cells continue producing IA for a longer duration, when in situ adsorption is performed. However, *U. cynodontis* IA_max_ is under stress and converts decreasing amounts of glucose into IA, limiting the described yield increase.

Compared to the experiments of Hosseinpour Tehrani et al. [19], yields in this work were increased at the cost of a decreased space–time yield. Increasing the yield directly improves the process from an economical point of view, as substrate consumption, presenting one of the main cost factors of the process, can be reduced [49]. To further improve the yield, the initially supplied amount of nitrogen can be reduced, further shifting the IA–biomass ratio at the cost of reduced productivity and space–time yield. Moreover, IA production can possibly be improved by feeding nitrogen at limiting concentrations during the production phase, to maintain a productive biocatalyst. An exemplary process for co-feeding of nitrogen and carbon source in the form of complex substrates for the production system in this thesis is described by Niehoff et al. [50]. The use of cheap waste streams from industry in their work is another possibility to make IA production with *U. cynodontis* IA_max_ economically more feasible.

The presented method is suitable for an implementation in larger scale. While adsorption columns can be scaled up based on the experience with the application of chromatography in industrial processes, the presented method for monitoring of metabolite concentrations with Raman spectroscopy can be used in other scales without elaborate adjustments. Most challenging is the implementation of the membrane filtration in larger scales. The use of an external membrane seems to be most suitable, posing additional future research questions, regarding the oxygen supply and hydrodynamic stress for the cells in an external loop.

## Conclusions

In this work, an in situ adsorption process with Raman online monitoring was successfully integrated into the production of IA with *Ustilago cynodontis* IA_max_, resulting in an improvement of titre, yield and space–time yield of the bioprocess, compared to the initial extended batch experiment without in situ product removal. The presented recovery method of adsorption and desorption with ethanol was performed at ambient temperatures and resulted in no additional formation of waste salts. A subsequent evaporation crystallisation of IA from the ethanolic product solution is connected to lower energy demands than rectification or direct crystallisation from the aqueous fermentation broth. In this first extended batch experiment, sequentially decreasing feed rates were introduced to ensure a low glucose concentration throughout the entire feed phase. This resulted in an increase of the selectivity of the planned in situ product recovery and limited the formation of the by-product erythritol. Investigations of the impact of the pH value on the adsorption capacity from mixtures of glucose and IA showed good selectivities at acidic pH values. After design of a complex process setup with five phases for repeated product separation, ethanol was introduced into the fermenter during a first proof of concept fermentation, caused by too short cycle times. In a next experiment, two cycles of in situ product separation via adsorption were conducted, separating around 10 g of IA and increasing the total titre to 84 g/L. For the final in situ experiment, a larger adsorption column was used to increase the adsorption capacity and, thereby, the amount of separated IA per cycle. In addition, Raman online monitoring was integrated, to improve process control of the glucose feed and switching between the steps of the separation cycles. In this optimised process, around 100 g of IA with a purity of above 95% *w*/*w* were separated by in situ adsorption without any ethanol introduction into the fermenter, resulting in a yield, titre and space–time yield of 0.41 g_IA_/g_Glucose_, 126.5 g_IA_ and 0.52 g_IA_/L/h. Compared to the initial extended batch experiment this is an increase in KPIs by up to 30%. Raman measurements predicted glucose and IA concentrations very accurately over nearly the whole process time, showing the immense value of this technique for bioprocess monitoring.

In conclusion, the presented process highlights the great potential of hydrophobic adsorption as an energy-efficient technique for direct recovery of non-polar products from bioprocesses.

### Supplementary Information

**Additional file1: Figure S1.** The IHM constructed for IA dissociation with glucose, ethanol, erythritol and water solvent. **Figure S2.** The IHM model fitted to a Raman spectrum at 0h recorded inline to the fermenter (Raman Sensor 1). **Figure S3.** The IHM model fitted to a Raman spectrum at 233.3 h recorded inline to the fermenter (Raman Sensor 1). **Figure S4.** Raman spectra recorded during the fermentation displayed in Fig. 7 of the manuscript. **Figure S5.** Raman spectra recorded during the extraction cycle between 87.4 and 99.5 h during the fermentation displayed in Fig. 7 of the manuscript. **Figure S6.** Raman spectra recorded during the extraction cycle between 88.9 and 95.4 h during the fermentation displayed in Fig. 7 of the manuscript. **Figure S7.** Process setup for *in situ *adsorption from extended batch fermentations. **Figure S8. **Extended batch cultivation of *U. cynodontis Δfuz7r Δcyp3r petefmtta pria1ria1 *in a 2 L fermenter with 100 g/L initial glucose and sequentially decreasing glucose feed rates. **Figure S9. **Chromatogram of product solution from desorption step of the second product separation cycle in experiment 4 (Fig. 7, 87.4–99.5 h). **Table S1.** Figures of merit for the total mixture hard model used with Raman spectroscopy.

## Data Availability

The datasets supporting the conclusions of this article are included within the article or the additional file (Additional file 1: Figures S1–S9, Table S1).

## Abbreviations

CDW: Cell dry weight
CTR: Carbon dioxide transfer rate
DI-water: Deionised water
DOT: Dissolved oxygen tension
H_2_IA: Fully protonated itaconic acid
HIA^−^: Single protonated itaconic acid
HPLC: High-performance liquid chromatography
IA: Itaconic acid
IA^2^^−^: Fully dissociated itaconic acid
KPI: Key performance indicator
OD_600_: Optical density at 600 nm
OTR: Oxygen transfer rate
RAMOS: Respiration activity monitoring system
RQ: Respiratory quotient
*U. cynodontis* IA_max_: *Ustilago cynodontis* NBRC9727 *Δfuz7*^*r*^* Δcyp3.*^*r*^* P*_*etef*_*mttA P*_*ria1*_*ria1*
