# Supplementary material for: In situ adsorption of itaconic acid from fermentations of Ustilago cynodontis improves bioprocess efficiency
Source: Biotechnol Biofuels Bioprod. 2023 Nov 26;16:181. doi: 10.1186/s13068-023-02433-w (PMC10676596; doi:10.1186/s13068-023-02433-w)
Supplement: Supplementary file 1 — Additional file1: Figure S1. The IHM constructed for IA dissociation with glucose, ethanol, erythritol and water solvent. Figure S2. The IHM model fitted to a Raman spectrum at 0h recorded inline to the fermenter (Raman Sensor 1). Figure S3. The IHM model fitted to a Raman spectrum at 233.3 h recorded inline to the fermenter (Raman Sensor 1). Figure S4. Raman spectra recorded during the fermentation displayed in Fig. 7 of the manuscript. Figure S5. Raman spectra recorded during the extraction cycle between 87.4 and 99.5 h during the fermentation displayed in Fig. 7 of the manuscript. Figure S6. Raman spectra recorded during the extraction cycle between 88.9 and 95.4 h during the fermentation displayed in Fig. 7 of the manuscript. Figure S7. Process setup for in situ adsorption from extended batch fermentations. Figure S8. Extended batch cultivation of U. cynodontis Δfuz7r Δcyp3r petefmtta pria1ria1 in a 2 L fermenter with 100 g/L initial glucose and sequentially decreasing glucose feed rates. Figure S9. Chromatogram of product solution from desorption step of the second product separation cycle in experiment 4 (Fig. 7, 87.4–99.5 h). Table S1. Figures of merit for the total mixture hard model used with Raman spectroscopy. [file 13068_2023_2433_MOESM1_ESM.pdf]

Additional file 1

In situ Adsorption of Itaconic Acid from Fermentations of *Ustilago cynodontis* Improves Bioprocess Efficiency

Johannes Pastoors[1], Alexander Deitert[1], Carina Michel[1], Karsten Günster[1], Maurice Finger[1], Jordy Hofstede[2], Jeff Deischter[3], Andreas Biselli[4], Jörn Viell[2], Regina Palkovits[3], Andreas Jupke[4] and Jochen Büchs[1]*

[1]AVT – Biochemical Engineering, RWTH Aachen University, Forckenbeckstraße 51, 52074 Aachen, Germany

[2]AVT – Process Systems Engineering, RWTH Aachen University, Forckenbeckstraße 51, 52074 Aachen, Germany

[3]ITMC - Institute of Technical and Macromolecular Chemistry, RWTH Aachen University, Worringerweg 2, 52074 Aachen, Germany

[4]AVT – Fluid Process Engineering, RWTH Aachen University, Forckenbeckstraße 51, 52074 Aachen, Germany

*Corresponding author:

Prof. Dr.-Ing. Jochen Büchs (Jochen.buechs@avt.rwth-aachen.de), AVT – Biochemical Engineering, RWTH Aachen University, Forckenbeckstraße 51, 52074 Aachen, Germany, Phone: +492418024633

Fig. SI 1. The IHM constructed for IA dissociation with glucose, ethanol, erythritol and water solvent.

Fig. SI 2. The IHM model fitted to a Raman spectrum at 0h recorded inline to the fermenter (Raman Sensor 1).

Fig. SI 3. The IHM model fitted to a Raman spectrum at 233.3h recorded inline to the fermenter (Raman Sensor 1).

Fig. SI 4. Raman spectra recorded during the fermentation displayed in Figure 7 of the manuscript.

Fig. SI 5. Raman spectra recorded during the extraction cycle between 87.4 and 99.5 h during the fermentation displayed in Figure 7 of the manuscript.

Fig. SI 6. Raman spectra recorded during the extraction cycle between 88.9 and 95.4 h during the fermentation displayed in Figure 7 of the manuscript.

Fig. SI 7. Process setup for *in situ* adsorption from extended batch fermentations.

Fig. SI 8. Extended batch cultivation of *U. cynodontis Δfuz7*^{r} *Δcyp3*^{r} p_{etef}*mtta* p_{ria1}*ria1* in a 2 L fermenter with 100 g/L initial glucose and sequentially decreasing glucose feed rates.

Fig. SI 9. Chromatogram of product solution from desorption step of the second product separation cycle in experiment 4 (Figure 7, 87.4-99.5 h).

Table SI 1. Figures of merit for the total mixture hard model used with Raman spectroscopy.

Indirect hard modelling (IHM) of Raman spectra

The IHM model used to determine the concentrations of glucose, IA and ethanol was constructed in PEAXACT and is shown in Fig. SI 1. It consists of three dissociated IA pure component models (PCMs) as described in Echtermeyer et al. and Saur, Kiefel, Niehoff et al. [1, 2]. Additional PCMs were created for glucose and ethanol, using 10 wt% solutions in water and for erythritol, using a 100 g/L solution in water. Furthermore, an auxiliary PCM was added to account for a nonlinear baseline due to underlying fluorescent signal that can overlay Raman spectra during biological processes. This fluorescence contribution is mostly an effect of growth and conversion by the microorganism and cannot be avoided.

The total mixture hard model is then calibrated with mixtures of glucose and ethanol between 1 and 10 wt% and itaconic acid in water between 0 and 8 wt%, respectively. The erythritol PCM is not calibrated and serves as a dummy model to ensure a correct fit of the major components, similar to the auxiliary fluorescence PCM.

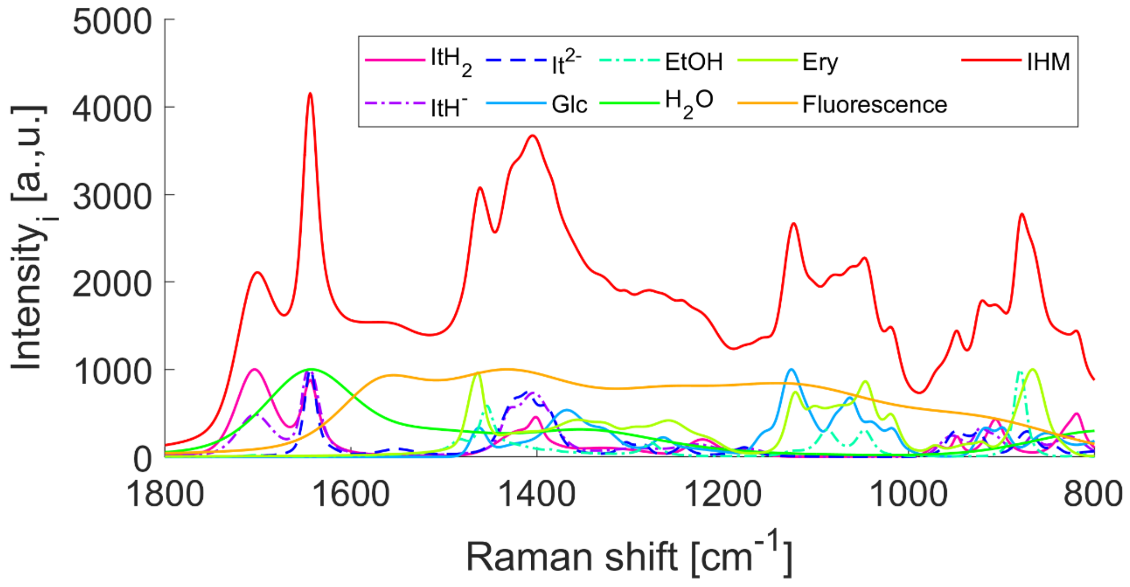

Fig. SI 1: The IHM constructed for IA dissociation with glucose, ethanol, erythritol and water solvent.

ItH_2: undissociated, ItH^-: first dissociation state and It^{2-}: fully dissociated IA, GLC: glucose, Ery: Eryhtritol, EtOH: Ethanol.

When fitting the Raman spectra, first a pretreatment model is applied, which excludes the oxygen gas peak at 1545-1565 1/cm. The spectral range of the model is reduced to 800-1800 1/cm and a linear baseline model is applied. The overall IHM then consists of PCMs that contain 18, 13 and 20 component peaks for glucose, ethanol and erythritol, respectively. The IA PCMs consist of 13, 14 and 16 component peaks for IAH_2, IAH^- and IA^{2-}, respectively. For fitting the weights of the PCMs, the two baseline parameters and the peak positions of each of the component peaks for all PCMS are flexible. The latter of which is allowed to vary by ±50 1/cm. For producing the fit, only 25 peaks are taken into account, which avoids extensive calculation times.

The model quality and accuracy are determined using a number of figures of merit. The performance of the calibration for component i is given by the root mean squared error of calibration ($RMSEC_i$) [g/g] and the root mean squared error of leave-10%-out cross-validation ($RMSECV_i$) [g/g], which are calculated as in (1):

$$RMSECV_i = \sqrt{(\frac{1}{K} \cdot \sum_{k=1}^{K} \left(w_{predicted,k,i} - w_{true,k,i}\right)^2} \quad (1)$$

Here, K is the total number of data points k used during calibration for a component i and w is the weight fraction in g/g. The RMSECV is obtained as a leave-10%-out cross-validation by the calibration algorithm which repeats the calibration several times, leaving out 10% of the data at every iteration until each data point is left out of the calibration at least once. The final RMSECV is obtained as the average of the iterations tested. The coefficient of determination, R^2_i, is used to measure the amount of variance in the true weight fractions explained by the predicted weight fractions.

Finally, a limit of detection is determined using (2) by evaluating 10 blank measurements of deionized water measured with Raman. The IHM model is used to calculate the weight fraction,

$w_{i,blank}$ and the standard deviation, $\sigma_{i,blank}$ of the blank measurements. A confidence factor, β, of 3 ensures that 99.86% confidence is obtained assuming normally distributed errors [3]:

$$LOD_i = \bar{w}_{i,blank} + \beta \cdot \sigma_{i,blank} \quad (2)$$

Except for water and ethanol, the $RMSEC_i$ and $RMSECV_i$ values are below 1.49 x 10^{-3} g/g, which indicates that the model has a good accuracy (**Fehler! Verweisquelle konnte nicht gefunden werden.**). The limit of detection (LOD_i) is around the same order of magnitude as the $RMSECV_i$'s, thus indicating precise predictions even at the low concentration range. The components in the model can thus be detected with an error of ±0.1 wt% and can be confidently detected at concentrations above 0.3 wt%.

Table SI 1: Figures of merit for the total mixture hard model used with Raman spectroscopy.

	R^2_i	$RMSEC_i$	$RMSECV_i$ 1)	LOD_i 2)
	[-]	[x10^{-3} g/g]	[x10^{-3} g/g]	[x10^{-3} g/g]
ItH2	0.999	0.576	0.611	0.651
ItH	0.993	1.139	1.146	0.424
It	0.998	0.505	0.517	0.348
H2O	0.995	1.415	1.493	n.d. 3)
GLC	0.9995	0.477	0.544	3.049
ETOH	0.9999	0.254	0.261	<0.01

1. Root mean squared error of leave-10%-out cross-validation
2. LOD = Limit of detection
3. n.d. = not defined

The calibrated IHM model is then used for prediction Raman spectra recorded inline during the fermentation and adsorption process. Fig. SI 2 and Fig. SI 3 show a Raman spectrum at the start and end of the fermentation displayed in Figure 7 of the manuscript, respectively. Both at the

start and end of the fermentation, the IHM produces a precise fit with the RMS residuals ($RMS = \sqrt{(\frac{1}{N} \cdot \sum_{n}^{N} \left(y_{model,n} - y_{measured,n}\right)^2)}$) around 2 orders of magnitude lower than the maximal spectral intensities observed. In addition, the residuals are randomly distributed across the width of the spectrum, thus providing a consistent result without missing components in the model.

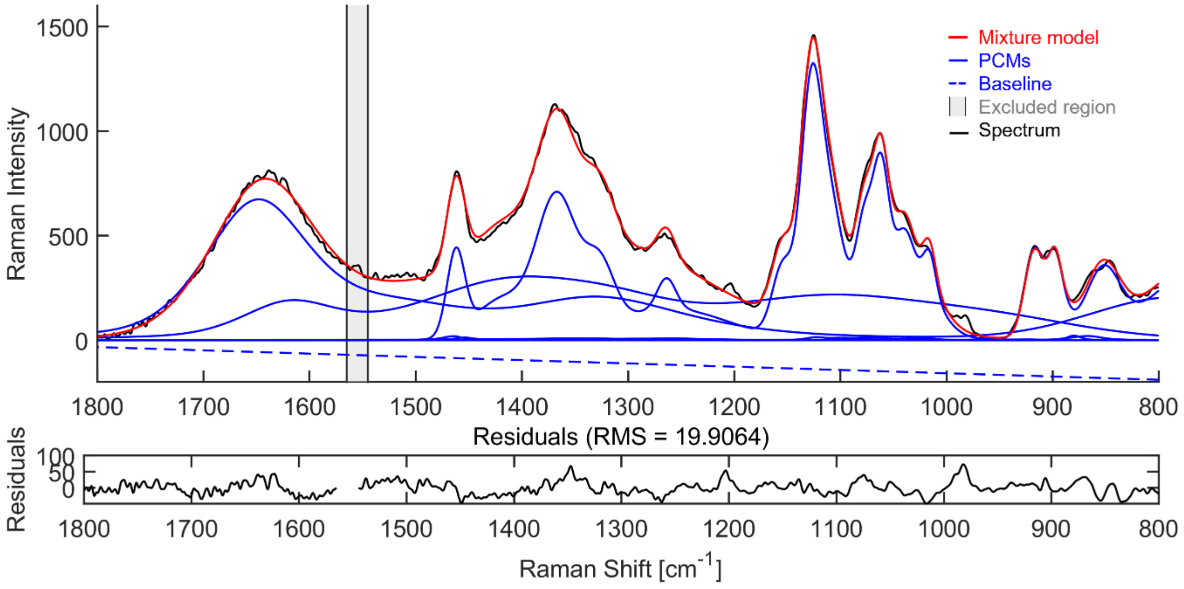

Fig. SI 2: The IHM model fitted to a Raman spectrum at 0 h recorded inline to the fermenter (Raman Sensor 1). The residuals indicate the root-mean squared difference between the fitted mixture model and recorded spectrum.

In Fig. SI 2, the main contribution to the observable peaks comes from glucose. Its most prominent bands are the δ (C-O-H) bending vibration at 1126 1/cm, the ν (C-O) stretching vibrations between 1000-1100 1/cm and the *w* (CH_2) wagging vibrations around 1300-1400 1/cm. In addition, the δ (H-O-H) water bending vibration can also be recognized at 1640 1/cm. It is also present in the spectrum at the end of the fermentation (Fig. SI 3), but there it overlaps with the ν (C=C) carbonyl stretching vibration of IA. A mixture of 2 species of IA, namely IAH_2 and IAH^- is observed as well, indicated by the 2 PCMs that have a peak at around 1700 1/cm. This mixture is the result of the acid dissociation equilibrium at the pH of approximately 3.6 at the end of the fermentation.

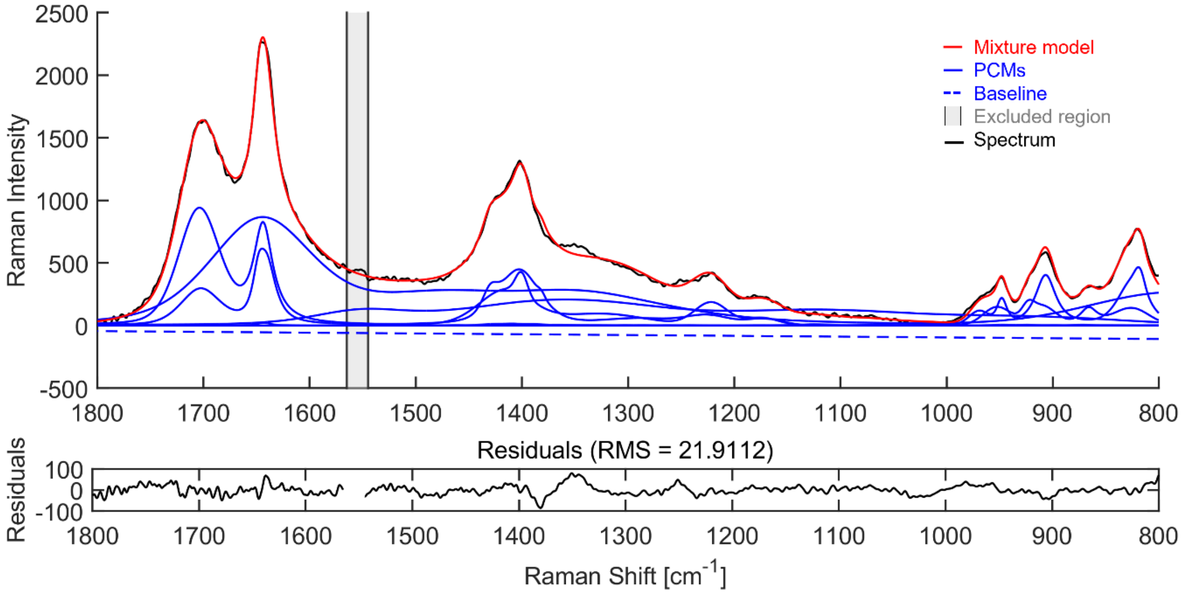

Fig. SI 3: The IHM model fitted to a Raman spectrum at 233.3 h recorded inline to the fermenter.

The residuals indicate the root-mean squared difference between the fitted mixture model and recorded spectrum.

Fig. SI 4 shows a subset (every 10th spectrum) of the total spectra recorded in Raman Sensor 1 during the fermentation displayed in Figure 7 of the manuscript. The large peak around 3500 1/cm is a result of the OH stretching vibration of water. Additionally, peaks resulting from other components are visible between 800 and 1800 1/cm. Between 70 and 150 h a regular fluctuation of the visible peaks below 2000 1/cm is visible, which is a result of repeated adsorption cycles to remove the produced itaconic acid from solution.

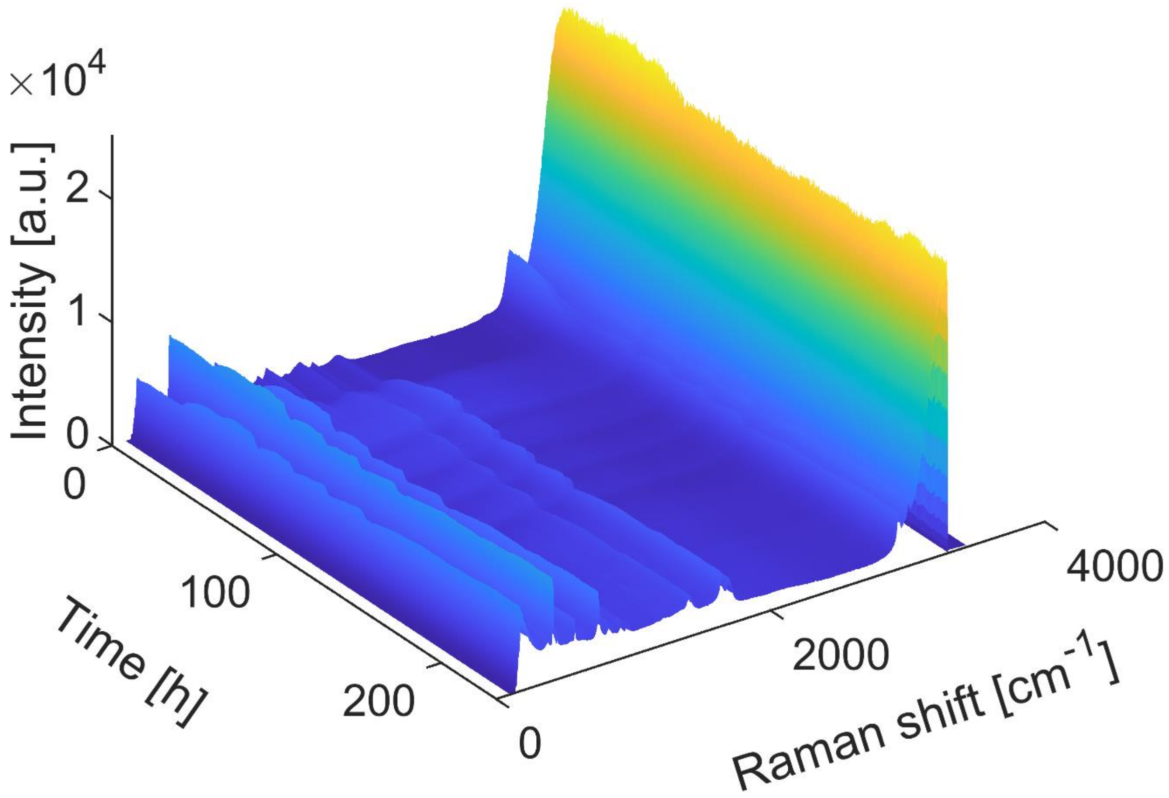

Fig. SI 4: Raman spectra recorded during the fermentation displayed in Figure 7 of the manuscript.

The large peak visible around 3500m 1/cm is a result of the (as)symmetric O-H stretching vibrations of water. In the region between 800 and 1850 1/cm also characteristic vibrations of other solutes are visible. Some fluctuations of component peak intensities can also be observed around 100 h which are the result of the adsorption cycles that remove mainly itaconic acid from solution.

The predictions shown in Figure 6 of the manuscript are determined from the Raman spectra collected in Raman Sensor 2 as shown in Fig. SI 5 and in more detail in Fig. SI 6. These were recorded between 87.4 and 99.5 h after the start of the fermentation process. Characteristic CH_3 stretching vibrations for ethanol can be observed in Fig. SI 5, while the OH stretching vibrations of water can also be observed in Fig. SI 6 after reducing the spectra to the time interval between 88.9 and 95.4 h.

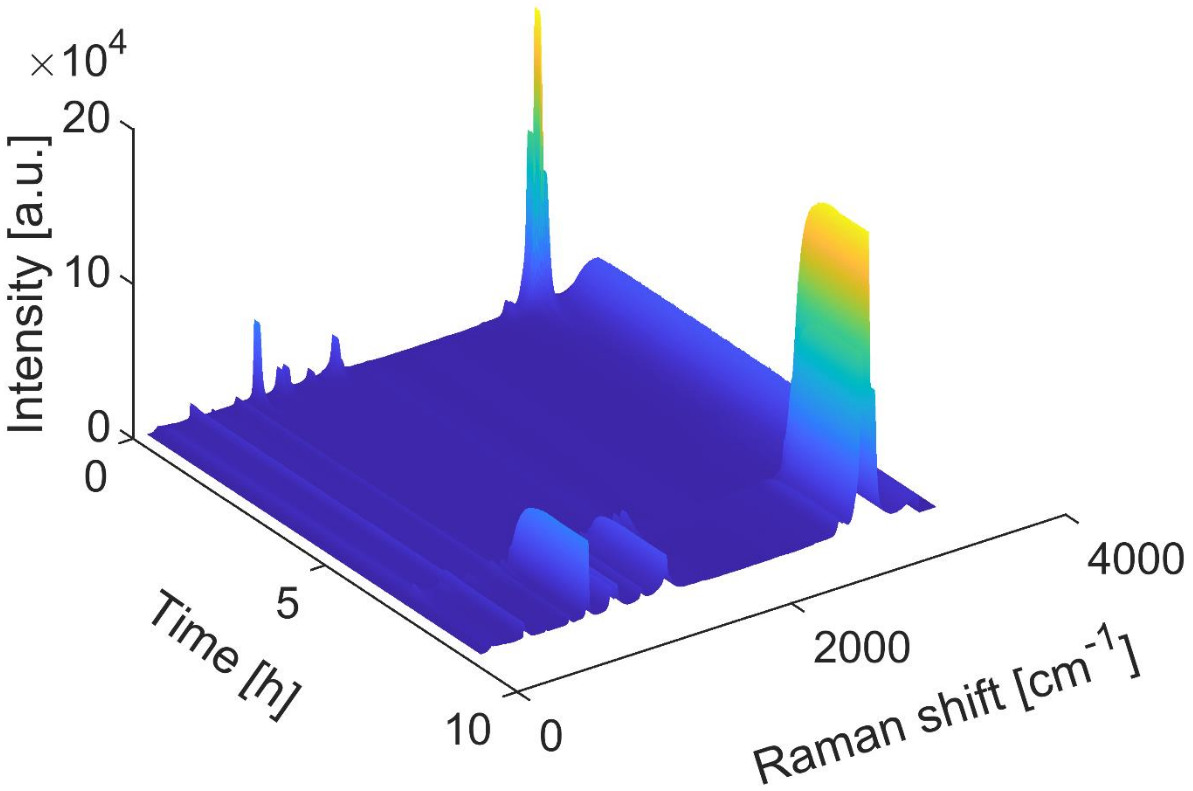

Fig. SI 5: Raman spectra recorded during the extraction cycle between 87.4 and 99.5 h during the fermentation displayed in Figure 7 of the manuscript.

The large peaks visible around 2934 1/cm are from (as)symmetric CH_3 stretching vibrations of ethanol. Due to Ethanol being very raman-active and present at high concentrations, the bands of other components in the range of 300-1850 1/cm are not observable.

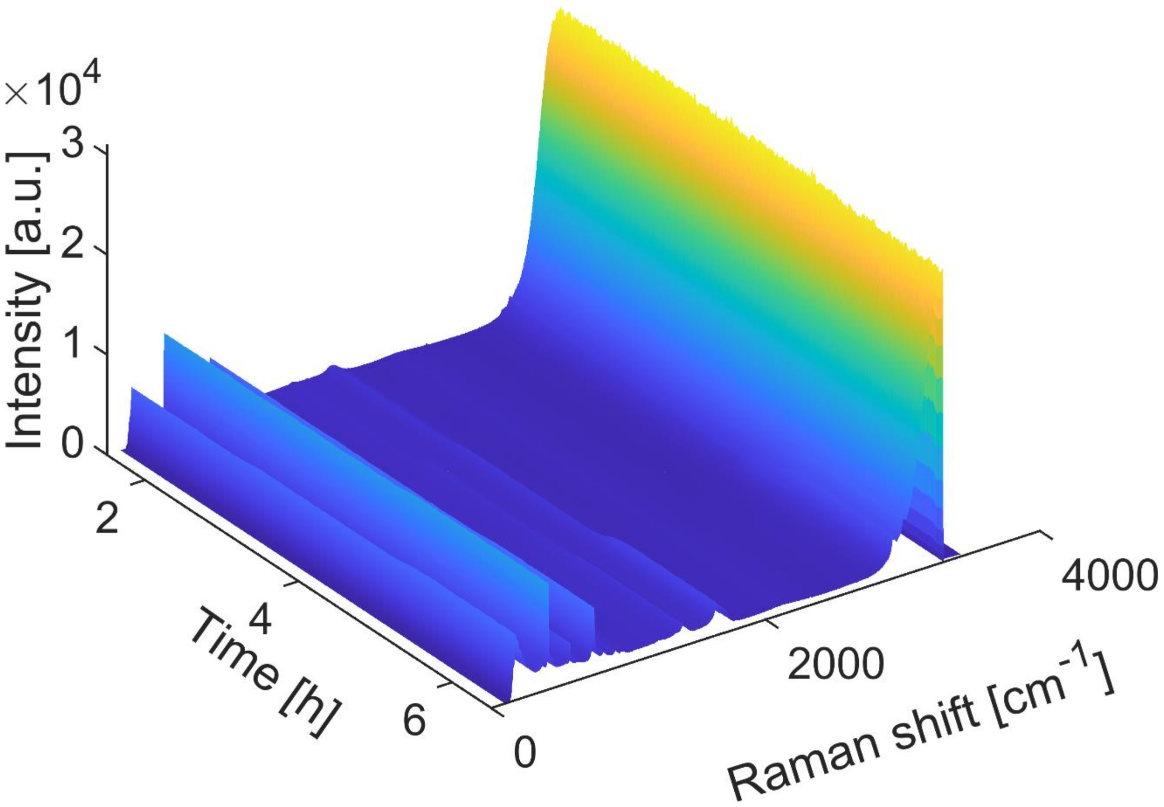

Fig. SI 6: Raman spectra recorded during the extraction cycle between 88.9 and 95.4 h during the fermentation displayed in Figure 7 of the manuscript.

Shown are the spectra that do not contain ethanol, to visualize the other components present in the spectra during the extraction. The large peak visible around 3500m 1/cm is a result of the (as)symmetric O-H stretching vibrations of water. In the region between 800 and 1850 1/cm also characteristic vibrations of other solutes are visible.

For transformation to volumetric concentrations, a correlation between the density of the fermentation supernatant δ_{Cycle} and the concentrations of IA c_{IA} and glucose $c_{Glucose}$ was used as displayed in (3):

$$\delta_{Cycle} = 1 + c_{Glucose} \cdot 0.000411 + c_{IA} \cdot 0.000506 \quad (3)$$

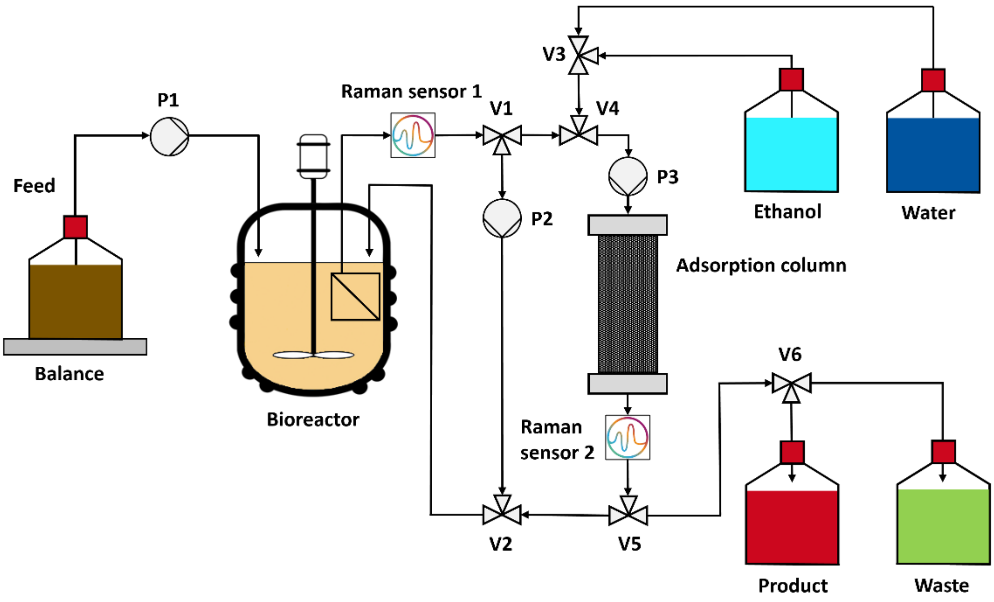

Fig. SI 7: Process setup for *in situ* adsorption from extended batch fermentations.

The weight of glucose fed into reactor by pump P1 is monitored by a balance. A stream of cell-free fermentation broth into the loop is ensured by pumps P2 or P3 applying underpressure on the permeate side of the hollow-fiber membrane in the reactor. Flow directions during different steps**:**

Fermentation: Bioreactor → Raman sensor 1 → V1 → P2 → V2 → Bioreactor

Washing: Water tank → V3 → V4 → P3 → Adsorption column → Raman sensor 2 → V5 → V6 → Waste tank + **Fermentation step**

Water displacement: Bioreactor → Raman sensor 1 → V1 → V4 → P3 → Adsorption column → Raman sensor 2 → V5 → V6→ Waste tank

Adsorption: Bioreactor → Raman sensor 1 → V1 → V4 → P3 → Adsorption column → Raman sensor 2 → V5 → V2 → Bioreactor

Broth displacement: Ethanol tank → V3 → V4 → P3 → Adsorption column → Raman sensor 2 → V5 → V2 → Bioreactor

Desorption: Ethanol tank → V3 → V4 → P3 → Adsorption column → Raman sensor 2 → V5 → V6 → Product tank + **Fermentation step**

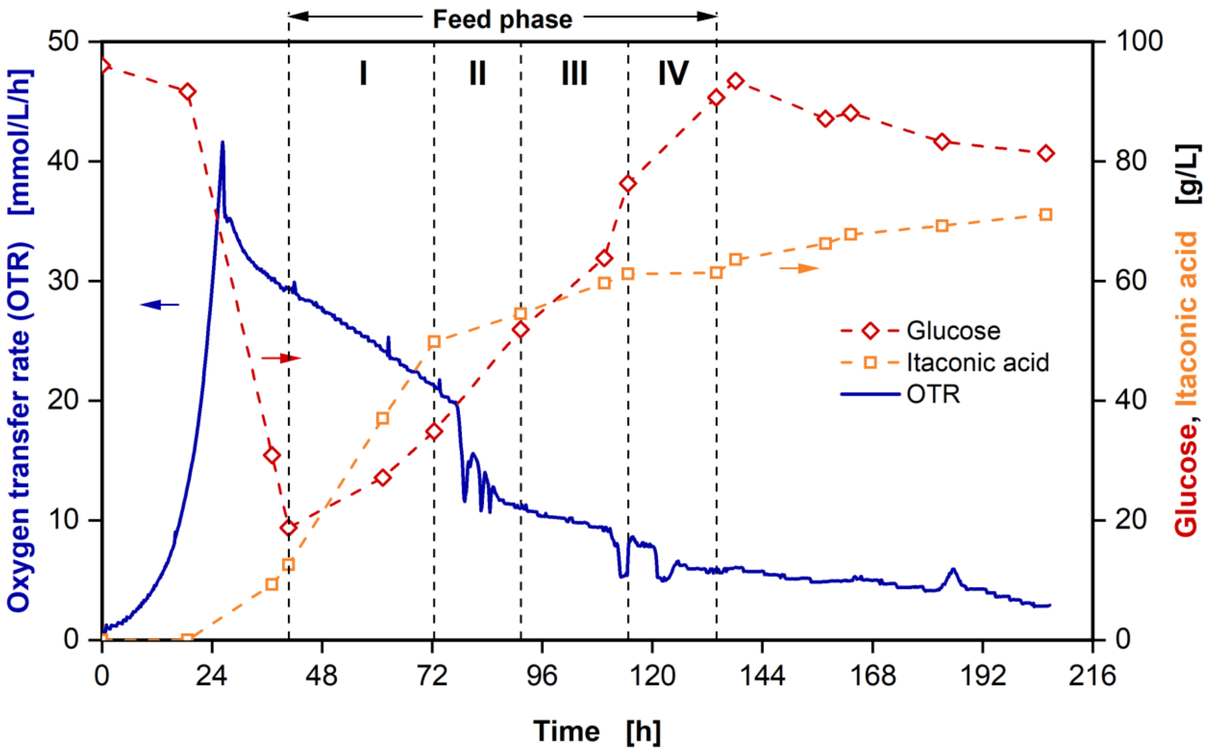

Fig. SI 8: Extended batch cultivation of *U. cynodontis* Δ*fuz7*^{r} Δ*cyp3*^{r} p_{etef}*mtta* p_{ria1}*ria1* in a 2 L fermenter with 100 g/L initial glucose and sequentially decreasing glucose feed rates.

Depicted are oxygen transfer rate (OTR) and concentrations of glucose and itaconic acid. Cultivation was performed at T = 30 °C, n = 300-560 rpm, $pH_{initial}$ = 6.3, $pH_{control}$ = 3.6 (10 M NaOH), $OD_{600,initial}$ = 0.5 and $V_{L,initial}$ = 1250 mL in adapted Verduyn medium in a 2 L fermenter. Aeration rate adjusted at beginning of each feed phase, to reach a mean volume specific aeration rate of q_{in} = 60 SL/L/h. Glucose was fed from a 519.0 g/L solution, resulting in feed rates of I: 4.1 g/h, II: 3.6 g/h, III: 3.0 g/h and IV: 2.4 g/h. For concentrations, mean values of two replicates are shown.

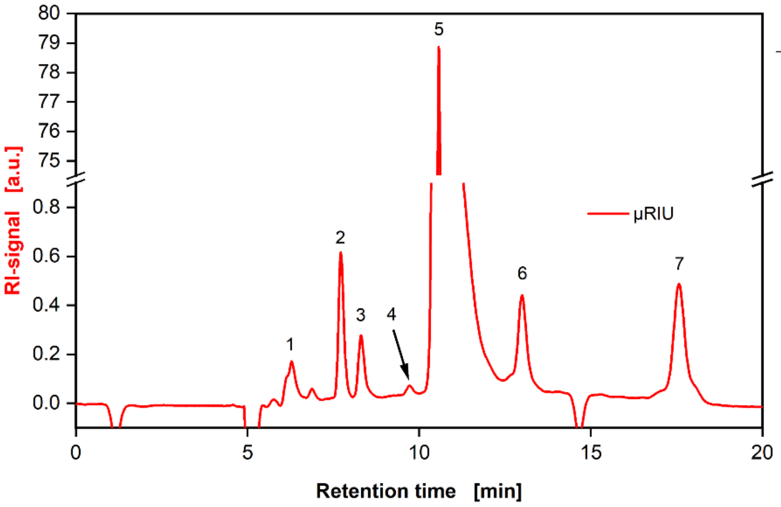

Peak-No.	Substance	Concentration
1	Unknown	Unkown
2	Glucose	0.02 g/L
3	Unknown	Unknown
4	Erythritol	0.00 g/L
5	Itaconic acid	6.30 g/L
6	Unknown	Unknown
7	Unknown	Unknown

Fig. SI 9: Chromatogram of product solution from desorption step of the second product separation cycle in experiment 4 (Figure 7, 87.4-99.5 h).

The RI-signal is depicted over the retention time. Ethanol from the product solution was evaporated. The remaining crystals were dissolved in DI-water before dilution, filtration and measurement.

References

[1] Saur, K., Kiefel, R., Niehoff, P.-J., Hofstede, J. et al., Holistic Approach to Process Design and Scale-Up for Itaconic Acid Production from Crude Substrates. *Bioengineering*, 2023(10).

[2] Echtermeyer, A., Marks, C., Mitsos, A., and Viell, J., Inline Raman spectroscopy and indirect hard modeling for concentration monitoring of dissociated acid species. *Applied Spectroscopy*, 2021, 75(5):506–519.

[3] El-Didamony, A.M. and Abo-Elsoad, M.O., Application of Methyl Orange in the Spectrophotometric Determination of Citalopram and Dapotextine in Pharmaceutical Formulations. *Journal of Applied Pharmaceutical Science*, 2016, 12(6):151–156.
